# Rosmarinic Acid Enhances Doxorubicin Activity in MDA-MB-231 Cells: Associations with Intracellular Oxidative Stress, Mitochondrial Dysfunction, and Apoptosis

**DOI:** 10.3390/biomedicines14091977

**Published:** 2026-09-02

**Authors:** Coşkun Orhaner, Mehmet Cudi Tuncer, İlhan Özdemir

**Affiliations:** 1 Department of Gynecology and Obstetrics, Soranus IVF Center, Bursa 16070, Turkey; orhanercoskun@gmail.com; 2 Department of Anatomy, Faculty of Medicine, Dicle University, Diyarbakır 21280, Turkey; 3 Department of Histology and Embryology, Faculty of Medicine, Kahramanmaraş Sütçü İmam University, Kahramanmaraş 46000, Turkey; ilhanozdemir32@hotmail.com

**Keywords:** Rosmarinic acid, breast cancer, synergism, apoptosis, mitochondrial pathway, Caspase-9

## Abstract

**Background/Objectives:** Rosmarinic acid (RA) is a naturally occurring polyphenolic compound with promising anticancer activity; however, its potential to enhance the therapeutic efficacy of doxorubicin (DOX) against triple-negative breast cancer (TNBC) has not been comprehensively characterised. This study investigated the pharmacological interaction and associated cellular responses of RA combined with DOX in MDA-MB-231 breast cancer cells, while including HaCaT human keratinocytes as a non-malignant, non-mammary reference model for comparative cytotoxicity assessment. **Methods:** Cell viability was assessed using the MTT assay, and pharmacological interactions were evaluated using Chou–Talalay combination index (CI) and dose reduction index (DRI) analyses. Intracellular oxidative activity was evaluated using DCFH-DA fluorescence analyses together with N-acetyl-L-cysteine (NAC) modulation experiments. Apoptosis, cell-cycle distribution, mitochondrial membrane potential (JC-1), Caspase-9 immunocytochemistry, nuclear morphology (NucBlue staining), apoptosis- and proliferation-related gene expression (RT-qPCR; *BAX*, *BCL2*, *CASP3*, *CASP9*, *TP53*, *CDKN1A*, *MKI67*, and *PCNA*), and bioinformatic pathway analyses were performed to characterise cellular and molecular responses associated with the combined treatment. **Results:** After 48 h, RA exhibited IC_50_ values of 188.4 ± 4.2 µM in MDA-MB-231 cells and 146.6 ± 6.8 µM in HaCaT cells, while DOX showed IC_50_ values of 1.2 ± 0.08 µM and 2.6 ± 0.15 µM, respectively. The Chou–Talalay analysis demonstrated synergistic interactions in MDA-MB-231 cells, with CI values of 0.86, 0.72, and 0.64 at the effect levels of IC_25_, IC_50_, and IC_75_, respectively; the model-derived DOX DRI value at Fa = 0.50 was 1.8. Combination treatment markedly increased intracellular DCFH-DA fluorescence, whereas NAC pretreatment attenuated this signal and partially restored cell viability, supporting a contributory role of intracellular oxidative stress in treatment-associated cytotoxicity. Increased intracellular oxidative activity coincided with mitochondrial membrane depolarisation, increased apoptotic cell death, accumulation of cells in the sub-G_1_ phase, enhanced Caspase-9 immunoreactivity, and pronounced apoptotic nuclear alterations. RT-qPCR analysis demonstrated significant upregulation of *BAX*, *CASP3*, *CASP9*, and *TP53*, together with downregulation of *BCL2*, *MKI67*, and *PCNA*, resulting in a marked reduction in the *BCL2*/*BAX* mRNA expression ratio. A modest but non-significant increase in *CDKN1A* expression was also observed. Bioinformatic analyses further identified predicted associations with mitochondrial apoptosis and p53-associated signalling pathways. **Conclusions:** The RA + DOX combination showed synergistic cytotoxic activity in MDA-MB-231 cells, accompanied by increased intracellular oxidative activity, mitochondrial membrane depolarisation, apoptosis, cell-cycle perturbation, and changes in proliferation-related gene expression. These findings indicate associated cellular responses but do not establish a ROS-dependent mitochondrial apoptotic mechanism. The findings provide an exploratory in vitro basis for further investigation of the RA + DOX combination in additional TNBC models, non-malignant mammary epithelial cells, and appropriate in vivo systems.

## 1. Introduction

Breast cancer remains the most frequently diagnosed malignancy among women and is responsible for a substantial proportion of the global cancer burden, accounting for approximately 12% of all newly diagnosed cancers worldwide [1]. Although remarkable advances have been achieved in systemic chemotherapy, the long-term clinical efficacy of current treatment regimens is frequently compromised by acquired drug resistance and dose-dependent toxicities, underscoring the need for more effective and safer therapeutic approaches [2]. Consequently, increasing attention has been directed toward combining conventional anticancer agents with naturally derived bioactive compounds to enhance therapeutic efficacy while minimizing adverse effects and reducing the required doses of cytotoxic drugs [3,4].

DOX, an anthracycline-based chemotherapeutic agent, constitutes a key component of treatment protocols for breast cancer as well as several solid and hematologic malignancies [5]. Its antitumor activity is primarily attributed to DNA intercalation, inhibition of topoisomerase II, and induction of oxidative stress through excessive intracellular ROS generation, ultimately triggering oxidative DNA damage, mitochondrial dysfunction, and apoptotic cell death [6]. Nevertheless, the clinical application of DOX is substantially restricted by cumulative cardiotoxicity, myelosuppression, and the emergence of multidrug resistance, all of which contribute to failure of treatment and limit its therapeutic benefit [7].

RA (RA; 3,4-dihydroxycinnamyl 3,4-dihydroxyphenyl acetate) is a naturally occurring polyphenolic ester abundantly present in medicinal and culinary plants of the Lamiaceae family, including rosemary (*Rosmarinus officinalis*) and sage (*Salvia officinalis*) [8]. Due to its diverse biological activities, RA has been extensively investigated as a multifunctional phytochemical with antioxidant, anti-inflammatory, antimicrobial and anticancer properties [9,10]. Accumulating experimental evidence indicates that RA has been reported to suppress tumor progression through multiple complementary mechanisms, including regulation of cell-cycle progression, induction of apoptosis, inhibition of angiogenesis, and modulation of intracellular signaling pathways involved in cancer cell survival and proliferation [11,12].

Recent advances in cancer pharmacology have increasingly supported the integration of naturally occurring polyphenols with established chemotherapeutic agents as a promising strategy to enhance antitumor efficacy while mitigating dose-dependent adverse effects. Among these compounds, RA has emerged as a potential chemosensitizing agent due to its ability to regulate multiple cellular processes involved in tumour progression and therapeutic resistance. Recent experimental evidence has demonstrated that RA significantly suppresses MDA-MB-231 triple-negative breast cancer cell proliferation, inhibits tumour spheroid growth, and induces mitochondrial apoptosis, further supporting its potential as an adjunct to conventional chemotherapy [13]. A comprehensive review published in 2024 summarized accumulating evidence demonstrating that RA potentiates the activity of several first-line anticancer drugs through modulation of oxidative stress, mitochondrial apoptosis, and signaling pathways associated with drug resistance, thereby highlighting its potential value as an adjunct to conventional chemotherapy. Furthermore, a recent review focusing specifically on triple-negative breast cancer emphasised that RA modulates apoptosis, cell-cycle progression, oxidative stress, and inflammatory signalling pathways involved in therapeutic resistance, supporting its development as a promising phytochemical chemosensitizing in breast cancer [14]. In addition, recent reviews focusing on breast cancer have emphasized that phytochemicals capable of concurrently regulating apoptosis, cell-cycle progression, cellular proliferation, and metastatic signaling may improve therapeutic responses while reducing systemic toxicity associated with standard chemotherapeutic regimens [12,15,16,17].

Despite these encouraging findings, the therapeutic interaction between RA and DOX has not yet been comprehensively investigated in TNBC. Existing studies have focused mainly on isolated biological endpoints, such as cytotoxicity or apoptosis, without integrating pharmacological synergy analyses with oxidative stress, mitochondrial dysfunction, mitochondrial membrane potential alterations, cell-cycle regulation, molecular alterations associated with apoptosis, and bioinformatic pathway analysis within a unified experimental framework. Consequently, the pharmacological interaction between RA and DOX and the cellular responses associated with their combined application in TNBC remain incompletely characterised. Addressing this gap may provide an experimental basis for evaluating this combination in more advanced preclinical models.

MDA-MB-231 cells were selected as the experimental model because they represent an aggressive subtype of TNBC characterised by high invasive capacity, metastatic behaviour, intrinsic resistance to multiple chemotherapeutic agents, and constitutive activation of several prosurvival signalling pathways [18]. These biological features closely resemble the clinical challenges associated with TNBC and make MDA-MB-231 cells an appropriate platform to investigate novel combination strategies designed to improve therapeutic efficacy and overcome drug resistance. HaCaT human keratinocytes were included as a non-malignant, non-mammary reference cell line to provide a limited comparative assessment of treatment-associated cytotoxicity. Because HaCaT cells are not derived from mammary epithelium, this comparison was not intended to establish tumour selectivity or define a therapeutic window [19].

Apoptosis is a tightly regulated form of programmed cell death that plays a fundamental role in tissue homeostasis and represents one of the principal mechanisms through which anticancer agents eliminate malignant cells [20]. Among apoptotic pathways, the intrinsic mitochondrial pathway is particularly important because it integrates intracellular stress signals and governs the activation of the caspase cascade through coordinated regulation of proteins from the Bcl-2 family [21]. Following mitochondrial outer membrane permeabilization, cytochrome c is associated with apoptotic protease-activating factor-1 and ATP to assemble the apoptosome, which subsequently activates Caspase-9 and initiates the downstream execution phase of apoptosis [22]. The balance between the pro-apoptotic protein Bax and the anti-apoptotic protein Bcl-2 is therefore considered a critical determinant of mitochondrial integrity and cellular susceptibility to apoptosis, with a reduced Bcl-2/Bax ratio that favours apoptotic cell death [23]. In addition, excessive intracellular ROS generation is closely related to mitochondrial dysfunction and can amplify intrinsic apoptotic signalling by promoting mitochondrial membrane depolarisation, providing an important mechanistic connection between oxidative stress and chemotherapy-induced cell death.

The present study was designed to evaluate whether RA enhances the cytotoxic activity of DOX in MDA-MB-231 cells and to characterise the associated changes in intracellular oxidative activity, mitochondrial function, apoptosis, cell-cycle distribution, and proliferation-related signalling. We hypothesised that the cellular response to combined RA and DOX exposure would be associated with increased intracellular oxidative stress, mitochondrial dysfunction, enhanced apoptosis, and suppression of proliferation-related processes, with intracellular oxidative stress potentially contributing to treatment-associated cytotoxicity. To evaluate this hypothesis, we employed an integrated experimental approach incorporating MTT-based cytotoxicity assays, Chou–Talalay pharmacological interaction analysis, DCFH-DA fluorescence analyses with NAC modulation experiments, Annexin V/PI apoptosis and cell-cycle flow cytometry, mitochondrial membrane potential assessment using JC-1, Caspase-9 immunocytochemistry, nuclear morphology assessment, RT-qPCR analysis of apoptosis- and proliferation-related genes, and bioinformatic pathway analyses.

Although previous studies have demonstrated the anticancer activity of RA as monotherapy and a limited number have evaluated RA-based combination strategies, the relationships among intracellular oxidative stress, mitochondrial dysfunction, apoptosis, and the cytotoxic response to combined RA and DOX exposure in TNBC have not been thoroughly characterised. The inclusion of intracellular oxidative activity, mitochondrial membrane potential, apoptosis, cell-cycle distribution, and gene-expression analyses enables a multidimensional assessment of the cellular events associated with the observed pharmacological interaction. This study therefore evaluates whether the increased cytotoxic activity observed following combined RA and DOX exposure is accompanied by alterations in intracellular oxidative activity, mitochondrial function, apoptosis, and proliferation-associated signalling. Beyond characterising the pharmacological interaction, the findings provide an exploratory framework for further investigation of these associated cellular responses in additional experimental models.

## 2. Materials and Methods

### 2.1. Culture and Routine Maintenance of the Experimental Cell Lines

Human triple-negative breast cancer MDA-MB-231 cells and HaCaT human keratinocytes were obtained from the American Type Culture Collection (ATCC, Manassas, VA, USA). MDA-MB-231 cells were maintained in RPMI-1640 medium, while HaCaT cells were cultured in Dulbecco’s Modified Eagle Medium. Both culture mediums were supplemented with 10% heat-inactivated fetal bovine serum, 100 U/mL penicillin, and 100 µg/mL streptomycin (all from Gibco, Thermo Fisher Scientific, Waltham, MA, USA). Cells were maintained at 37 °C in a humidified incubator containing 5% CO_2_. Culture media were renewed every 2–3 days, and cells were subcultured at approximately 80–90% confluence using 0.25% trypsin–EDTA (Gibco, Thermo Fisher Scientific). Experiments were performed using actively proliferating cells between passages 3 and 12. Before experimental use, cultures were examined by phase-contrast microscopy to confirm normal morphology, adequate adherence, and the absence of visible microbial contamination.

### 2.2. Preparation and Handling of RA and DOX Solutions

Rosmarinic acid (RA; purity ≥ 98%; Sigma-Aldrich, St. Louis, MO, USA) and doxorubicin hydrochloride (DOX; Sigma-Aldrich) were separately dissolved in DMSO to prepare concentrated stock solutions, which were diluted with the appropriate culture medium immediately before each experiment. The previously reported stock molarity was removed because it could not be independently verified from the available experimental records. The final DMSO percentage was verified independently of the nominal drug-stock molarity by calculating the volume of DMSO-containing stock solution added relative to the final volume of treatment medium. The maximum recorded stock-solution addition was 1 µL per 1 mL of treatment medium, corresponding to 0.1% (*v*/*v*) DMSO; smaller solvent volumes were used under the remaining treatment conditions. Corresponding vehicle-control groups containing the same final DMSO concentration as their respective treatment groups were included, and vehicle exposure did not measurably affect cellular morphology or viability. Fresh working solutions were prepared for each independent experiment.

### 2.3. Determination of Cytotoxic Activity and IC_50_ Values Using the MTT Colorimetric Assay

The cytotoxic effects of RA, DOX, and their combination were determined using the colorimetric MTT assay. MDA-MB-231 and HaCaT cells were placed in 96-well flat-bottom culture plates at a density of 5 × 10^3^ cells per well and allowed to adhere for 24 h under standard culture conditions. Following cell attachment, the culture medium was replaced with fresh medium containing serial concentrations of RA (10, 25, 50, 100, 250, 500, and 1000 µM), DOX (0.1, 0.5, 1, 2.5, 5, 10, and 25 µM), or their corresponding combination treatments. The untreated control and vehicle-control wells were included in each experiment, and all treatments were maintained for 48 h.

At the end of the exposure period, 20 µL of MTT solution (5 mg/mL in phosphate-buffered saline; Sigma-Aldrich, St. Louis, MO, USA) was added directly to each well, followed by incubation for an additional 4 h at 37 °C to allow viable cells to convert the tetrazolium salt into insoluble purple formazan crystals. The culture medium containing residual MTT reagent was then carefully aspirated and the resulting formazan crystals were completely dissolved in 200 µL of DMSO. Absorbance was measured at 570 nm using a microplate reader and background values obtained from the blank wells were subtracted before analysis. Cell viability was expressed as the percentage of the untreated control group, which was defined as 100%.

Dose–response curves were generated by nonlinear regression analysis using GraphPad Prism software (version 9.0; GraphPad Software Inc., San Diego, CA, USA), and the half-maximal inhibitory concentration (IC_50_) values for RA and DOX were calculated from three independent experiments.

### 2.4. Pharmacological Assessment of Drug Interactions Using the Chou–Talalay Method

The pharmacological interaction between RA and DOX was quantitatively evaluated using the Chou–Talalay median-effect principle implemented in CompuSyn software (version 1.0; ComboSyn Inc., Paramus, NJ, USA). This method is based on the median-effect equation derived from the mass-action law and enables quantitative assessment of drug interactions across a range of effect levels. Drug combinations were prepared at a fixed RA:DOX molar ratio of 157:1, derived from the respective IC_50_ values obtained in MDA-MB-231 cells (188.4 µM RA and 1.2 µM DOX). The fixed-ratio combination series comprised 47.1 µM RA + 0.30 µM DOX, 94.2 µM RA + 0.60 µM DOX, 188.4 µM RA + 1.20 µM DOX, 376.8 µM RA + 2.40 µM DOX, and 753.6 µM RA + 4.80 µM DOX. These paired concentrations corresponded to 0.25-, 0.5-, 1-, 2-, and 4-fold multiples, respectively, of the MDA-MB-231 IC_50_-based combination while maintaining the constant 157:1 molar ratio. The same fixed-ratio series was evaluated in both MDA-MB-231 and HaCaT cells to permit comparison of cell line-dependent interaction profiles.

Cell-viability data obtained from the MTT assay were converted to fraction-affected values, where Fa = 1 − the viable-cell fraction relative to the untreated control, and entered into CompuSyn to generate dose–effect curves and median-effect plots. The median-effect dose (Dm), slope of the median-effect plot (m), and linear correlation coefficient (r) were derived for each drug and cell model. Combination index (CI) values were calculated from the fitted median-effect model at Fa = 0.25, 0.50, and 0.75, corresponding to the IC_25_, IC_50_, and IC_75_ effect levels, respectively. These Fa levels were model-derived analytical points and did not represent separately administered treatment concentrations. Dose reduction index (DRI) values for DOX were evaluated at the median-effect level (Fa = 0.50) as model-derived estimates of the difference between the single-agent and combination doses predicted to produce the same effect.

The pharmacological interaction was interpreted according to the predefined Chou–Talalay criteria: CI values < 0.90 indicated synergism, values from 0.90 to 1.10 indicated an additive interaction, and values > 1.10 indicated antagonism. DRI values were interpreted descriptively and were not considered evidence of clinical dose reduction or therapeutic benefit. For subsequent cellular and molecular experiments, the 1-fold IC_50_-based combination consisting of 188.4 µM RA and 1.2 µM DOX was selected to produce measurable responses across the complementary assays. This concentration pair was not selected as a synergistic sub-IC_50_ regimen; instead, it was used to characterise the cellular responses associated with concurrent exposure to the individual IC_50_ concentrations. Accordingly, downstream mechanistic observations were not used to infer the mechanism responsible for the pharmacological synergism identified by the fixed-ratio analysis.

### 2.5. Assessment of Intracellular Reactive Oxygen Species by DCFH-DA Fluorescence Imaging, Flow Cytometry, and NAC Rescue

Intracellular oxidative changes were evaluated using 2′,7′-dichlorodihydrofluorescein diacetate (DCFH-DA; Sigma-Aldrich, St. Louis, MO, USA) through complementary fluorescence microscopy and flow cytometric analyses. Because DCFH-DA is a general oxidation-sensitive probe, this assay was used to assess general intracellular oxidative activity rather than mitochondria-specific ROS production. To investigate the contribution of oxidative stress to the biological effects of RA and DOX, intracellular ROS levels were quantified in the absence and presence of the antioxidant NAC.

For flow cytometric analysis, MDA-MB-231 cells were cultured in 6-well plates and exposed to RA (188.4 µM), DOX (1.2 µM), or their combination for 48 h. After treatment, the culture medium was removed and the cells were gently rinsed twice with phosphate-buffered saline (PBS) before incubation with 10 µM DCFH-DA in serum-free medium for 30 min at 37 °C in the dark. The cells were then washed with PBS, detached using trypsin–EDTA, collected by centrifugation, and immediately analysed using a BD FACSCanto II flow cytometer (BD Biosciences, San Jose, CA, USA). A minimum of 10,000 events was acquired for each sample. Cellular debris was excluded according to the forward scatter (FSC) and side scatter (SSC) characteristics, followed by doublet discrimination using FSC-A/FSC-H gating. Fluorescence signals were collected in the Fluorescein Isothiocyanate (FITC) channel using identical photomultiplier tube voltage settings for all samples. Intracellular ROS production was quantified from the mean fluorescence intensity (MFI) of DCF-positive cells, and all flow cytometric data were processed using BD FACSDiva software (version 8.0; BD Biosciences).

For fluorescence imaging, MDA-MB-231 cells were seeded onto sterile glass coverslips placed in 24-well plates at a density of 5 × 10^4^ cells per well and allowed to attach overnight before treatment with RA, DOX, or the combined regimen for 48 h. After drug exposure, cells were washed twice with PBS and incubated with 10 µM DCFH-DA for 30 min at 37 °C under light-protected conditions. After three additional PBS washes to remove excess probe, fluorescence images were acquired immediately using an inverted fluorescence microscope equipped with a FITC filter set. Exposure time, illumination intensity, objective magnification, camera gain, and all acquisition parameters were maintained identically throughout the image collection to permit direct comparison between experimental groups. Representative images were selected from three independent biological experiments. The acquisition of images and the quantitative analysis of the images were performed by an investigator blinded to the treatment groups.

Quantitative image analysis was performed using ImageJ software (version 1.54; National Institutes of Health, Bethesda, MD, USA). Following uniform background subtraction, the intracellular DCF fluorescence intensity was measured as MFI and normalised to the untreated control group, which was assigned a value of 1. Identical thresholding parameters and analysis settings were applied to every image to minimise operator-dependent variability. For each independent experiment, at least five randomly selected non-overlapping microscopic fields containing a minimum of 300 cells per treatment group were analysed.

To determine the functional contribution of oxidative stress to treatment-induced cytotoxicity, cells were pretreated with 5 mM NAC (NAC; Sigma-Aldrich, St. Louis, MO, USA) for 2 h before exposure to RA, DOX, or their combination. Following 48 h of treatment, intracellular ROS production was re-evaluated by DCFH-DA fluorescence microscopy and flow cytometry using the procedures described above. In parallel, cell viability was determined by the MTT assay. The effect of ROS scavenging was assessed by comparing intracellular ROS levels and cell viability between NAC-pretreated and non-pretreated groups, thus determining the extent to which attenuation of intracellular oxidative stress influenced the cytotoxic activity of RA, DOX, and their combined treatment.

### 2.6. Flow Cytometric Quantification of Apoptotic Cell Death

Apoptotic cell death was quantified using the FITC Annexin V Apoptosis Detection Kit I (BD Biosciences, San Jose, CA, USA) according to the manufacturer’s recommendations. Following completion of the respective treatments, both floating and adherent cell populations were collected to avoid underestimating apoptotic cells. Adherent cells were removed using trypsin–EDTA, combined with the corresponding floating cells, and washed twice with ice-cold PBS. The cell pellets were subsequently resuspended in 1× binding buffer to obtain a final concentration of approximately 1 × 10^6^ cells/mL.

For each sample, 5 µL of FITC-conjugated Annexin V and 5 µL of propidium iodide (PI) were added, followed by incubation for 15 min at room temperature in the dark. After staining, an additional binding buffer was added to each tube and samples were immediately analysed using a BD FACSCanto II flow cytometer (BD Biosciences, San Jose, CA, USA). A minimum of 10,000 events were acquired for each sample in identical instrument settings. Cellular debris was excluded according to the characteristics of FSC and SSC, and cell aggregates were removed by doublet discrimination of FSC a/FSC-H. Flow cytometric data were analysed using FlowJo software (version 10.0; BD Life Sciences, Ashland, OR, USA).

Cell populations were classified according to Annexin V and PI staining patterns as viable (Annexin V^−^/PI^−^), early apoptotic (Annexin V^+^/PI^−^), late apoptotic (Annexin V^+^/PI^+^), and necrotic (Annexin V^−^/PI^+^). Total apoptotic cells were calculated as the sum of early and late apoptotic populations and used for subsequent statistical analyses.

### 2.7. Flow Cytometric Analysis of Cell-Cycle Distribution

Cell-cycle distribution was determined by flow cytometric measurement of cellular DNA content using PI staining. Following treatment with RA (188.4 µM), DOX (1.2 µM), or their combination for 48 h, MDA-MB-231 cells were harvested by trypsinization, washed twice with ice-cold phosphate-buffered saline (PBS), and fixed overnight in 70% (*v*/*v*) ice-cold ethanol at 4 °C. Fixed cells were subsequently washed with PBS to remove residual ethanol and incubated with a staining solution containing 50 µg/mL propidium iodide (PI; Sigma-Aldrich, St. Louis, MO, USA) and 100 µg/mL RNase A (Sigma-Aldrich, St. Louis, MO, USA) for 30 min at room temperature in the dark.

DNA content was analysed using a BD FACSCanto II flow cytometer (BD Biosciences, San Jose, CA, USA), and a minimum of 10,000 events were acquired for each sample using identical instrument settings. Cellular debris was excluded according to forward scatter (FSC) and side scatter (SSC) characteristics, followed by doublet discrimination using FSC-A/FSC-H gating. Flow cytometric data were analysed using FlowJo software (version 10.0; BD Life Sciences, Ashland, OR, USA). Cell populations were quantified according to DNA content and classified into the Sub-G_1_, G_0_/G_1_, S, and G_2_/M phases. The Sub-G_1_ fraction was interpreted as the population of cells exhibiting hypodiploid DNA content, consistent with apoptotic DNA fragmentation. Representative DNA-content histograms were generated for each experimental group, and quantitative data are presented as the percentage of cells in each phase of the cell cycle from three independent biological experiments (*n* = 3).

### 2.8. Flow Cytometric Evaluation of Mitochondrial Membrane Potential (ΔΨm)

Changes in ΔΨm were determined using the cationic fluorescent probe JC-1 (5,5′,6,6′-tetrachloro-1,1′,3,3′-tetraethylbenzimidazolylcarbocyanine iodide; Cayman Chemical, Ann Arbor, MI, USA), which selectively accumulates within polarised mitochondria. Following the respective treatments, MDA-MB-231 cells were harvested, washed twice with PBS, and incubated with 2 µM JC-1 working solution for 30 min at 37 °C under light-protected conditions. After staining, cells were washed once with PBS to remove excess dye and immediately subjected to flow cytometric analysis.

Fluorescence acquisition was performed using a BD FACSCanto II flow cytometer (BD Biosciences, San Jose, CA, USA), and a minimum of 10,000 events were collected for each sample in identical instrument settings. Cellular debris was excluded using FSC and SSC characteristics, followed by doublet discrimination using FSC-A/FSC-H gating. JC-1 monomers, indicative of mitochondrial membrane depolarisation, were detected in the FITC channel (green fluorescence), while JC-1 aggregates, representing intact mitochondrial membrane potential, were detected in the PE channel (red fluorescence). Flow cytometric data were analysed using FlowJo software (version 10.0; BD Life Sciences, Ashland, OR, USA).

Mitochondrial membrane integrity was expressed as the ratio of the red to green fluorescence intensity (PE/FITC). A reduction in this ratio was interpreted as mitochondrial membrane depolarisation and loss of ΔΨm, indicating impaired mitochondrial function consistent with the involvement of the intrinsic apoptotic pathway.

### 2.9. Immunocytochemical Detection of Cleaved Caspase-9-Associated Immunoreactivity

The cleaved Caspase-9-associated immunoreactivity was evaluated by immunocytochemistry following the respective treatments. MDA-MB-231 cells were cultured on sterile glass coverslips placed in 24-well plates and allowed to adhere overnight before drug exposure. At the end of the treatment period, cells were fixed with freshly prepared 4% paraformaldehyde for 20 min at room temperature and subsequently rinsed three times with PBS. Cellular membranes were permeabilized using 0.1% Triton X-100 prepared in PBS for 10 min, followed by incubation with 5% bovine serum albumin (BSA) in PBS for 1 h at room temperature to minimize nonspecific antibody binding.

The cells were then incubated overnight at 4 °C with Cleaved Caspase-9 (Asp330) (D2D4) Rabbit Monoclonal Antibody (#7237; Cell Signaling Technology, Danvers, MA, USA) diluted 1:200 in antibody diluent. According to the manufacturer, this antibody preferentially recognises Caspase-9 cleaved at Asp330, although weak detection of full-length Caspase-9 may occur in some cellular contexts. Following three PBS washing steps, the samples were incubated with a biotinylated secondary antibody for 1 h at room temperature and subsequently treated with a streptavidin–horseradish peroxidase detection system according to the manufacturer’s recommendations. Immunoreactivity was visualised using 3,3′-diaminobenzidine (DAB) chromogen, resulting in a brown reaction product, while the nuclei were counterstained with Mayer’s hematoxylin.

The stained preparations were examined with a light microscope under identical imaging conditions for all experimental groups. Cleaved Caspase-9-associated immunoreactivity was quantified using a semiquantitative H-score method. Cytoplasmic staining intensity was classified for each evaluated cell as 0 (no staining), 1 (weak staining), 2 (moderate staining), or 3 (strong staining). The percentage of cells within each staining-intensity category was determined, and the H-score was calculated using the formula H-score = Σ(P_i_ × i), where P_i_ represents the percentage of cells exhibiting staining intensity i and i represents the corresponding intensity category from 0 to 3. The resulting H-score ranged from 0 to 300.

For each independent biological experiment, at least five randomly selected non-overlapping microscopic fields and a minimum of 300 cells per treatment group were evaluated. Three independent biological experiments were analysed (*n* = 3), and one H-score value was calculated for each biological replicate. Image acquisition and H-score assessment were performed by an investigator blinded to the treatment groups. Representative micrographs were selected from the same independently analysed experiments. H-score values were statistically compared using one-way ANOVA followed by Tukey’s multiple-comparisons post hoc test. The immunocytochemical findings were interpreted as cleaved Caspase-9-associated immunoreactivity rather than definitive evidence of Caspase-9 activation.

### 2.10. Fluorescence-Based Assessment of Nuclear Morphological Alterations

Nuclear morphological changes associated with apoptosis were evaluated using NucBlue™ Live ReadyProbes™ Reagent (Thermo Fisher Scientific, Waltham, MA, USA). MDA-MB-231 cells were seeded onto sterile glass coverslips placed in 24-well plates and allowed to adhere for 24 h prior to treatment. The cells were then exposed to RA, DOX, or their combination for 48 h under standard culture conditions.

Following treatment, the culture medium was removed and the cells were gently washed twice with PBS. NucBlue™ Live ReadyProbes™ Reagent was added directly to the culture medium according to the manufacturer’s instructions, and cells were incubated at 37 °C under light-protected conditions. Fluorescence images were acquired immediately using an inverted fluorescence microscope equipped with a DAPI-compatible filter set. Exposure time, illumination intensity, objective magnification, camera settings, and all acquisition parameters were kept constant for all experimental groups to ensure direct comparison among samples.

The apoptotic nuclear morphology was qualitatively assessed by evaluating characteristic apoptotic features, including chromatin condensation, nuclear shrinkage, nuclear fragmentation, increased fluorescence intensity, and apoptotic body formation. In addition, a quantitative analysis was performed by determining the percentage of cells exhibiting apoptotic nuclear morphology. For each independent experiment, at least five randomly selected non-overlapping microscopic fields were analysed, and a minimum of 300 nuclei per treatment group were evaluated using ImageJ software (version 1.54; National Institutes of Health, Bethesda, MD, USA). Cells displaying chromatin condensation and/or nuclear fragmentation were classified as apoptotic, and the percentage of apoptotic nuclei was calculated relative to the total number of nuclei counted. Representative fluorescence images from three independent biological experiments were selected for presentation. Image acquisition and quantitative analysis were performed by an investigator blinded to the allocation of treatment.

### 2.11. RT-qPCR Analysis of Apoptosis- and Proliferation-Related Gene Expression

Following 48 h of treatment with RA (188.4 µM), DOX (1.2 µM), or their combination, total RNA was isolated from MDA-MB-231 cells using the PureLink RNA Mini Kit (Thermo Fisher Scientific, Waltham, MA, USA) according to the manufacturer’s instructions. RNA concentration and purity were assessed spectrophotometrically by measuring absorbance at 260 and 280 nm, and samples with A_260_/A_280_ ratios between 1.8 and 2.0 were used for subsequent analyses. One microgram of total RNA was reverse-transcribed into complementary DNA (cDNA) using the High-Capacity cDNA Reverse Transcription Kit (Applied Biosystems, Thermo Fisher Scientific) according to the manufacturer’s protocol.

Gene-expression analyses were performed using SYBR Green-based quantitative real-time PCR (RT-qPCR). Amplification reactions were prepared in 96-well plates using PowerUp SYBR Green Master Mix (Applied Biosystems, Thermo Fisher Scientific) in a final reaction volume of 10 µL. RT-qPCR analyses were carried out using a QuantStudio Real-Time PCR System (Applied Biosystems, Thermo Fisher Scientific). Cycling conditions consisted of an initial denaturation step at 95 °C for 10 min, followed by 40 amplification cycles of 95 °C for 15 s and 60 °C for 1 min.

Expression levels of the apoptosis-related genes *BAX*, *BCL2*, *CASP3*, *CASP9*, and *TP53*, the cell-cycle regulatory gene *CDKN1A*, and the proliferation-associated genes *MKI67* and *PCNA* were evaluated. *GAPDH* and *ACTB* were used as reference genes for normalisation, and the geometric mean of their expression levels was used for target-gene normalisation. Relative gene-expression levels were calculated using the 2^−ΔΔCt^ method, with the untreated control group used as the calibrator.

Melting-curve analysis was performed following each amplification run to verify amplification specificity. No-template controls and no-reverse-transcription controls were included to monitor reagent contamination and genomic DNA carryover, respectively. Primer sequences used in the study are presented in Table 1. Three independent biological experiments were performed (*n* = 3), and each biological sample was analysed in technical triplicate. Technical replicates were averaged within each biological replicate before statistical analysis.

### 2.12. Protein Interaction Network Construction and Functional Enrichment Analysis

To further explore the functional relationships among the apoptosis-related genes examined experimentally, an in silico bioinformatic analysis was performed. The RT-qPCR-assessed genes *BAX*, *BCL2*, *TP53*, *CDKN1A*, *CASP3*, and *CASP9* were used as the experimentally derived input set. *CYCS* was included solely as a pathway-associated contextual node to visualise its predicted interaction with the BAX/BCL2–Caspase-9 axis; *CYCS* expression and cytochrome-c release were not experimentally measured in the present study. The selected genes were imported into the STRING database (version 12.0; https://string-db.org; accessed on 1 July 2026) to construct a protein–protein interaction network and identify predicted functional associations among the encoded proteins. Protein interactions were analysed using a medium confidence interaction score of 0.400.

Functional enrichment analyses were subsequently performed using the Database for Annotation, Visualisation, and Integrated Discovery (DAVID; https://davidbioinformatics.nih.gov; accessed on 1 July 2026). Biological process analysis of gene ontology (GO) and Kyoto Encyclopaedia of Genes and Genomes (KEGG) pathways were conducted to identify biological functions and signalling pathways significantly enriched in the selected set of genes. Enriched terms with an italicised *p* value < 0.05 were considered statistically significant. Protein interaction networks and enrichment results were visualised using Cytoscape software (version 3.10.1; Cytoscape Consortium, Seattle, WA, USA).

### 2.13. Statistical Analysis

Unless otherwise stated, all quantitative experiments were performed as three independent biological experiments (*n* = 3). Where technical replicates were included, they were averaged within each biological replicate before statistical analysis. The results are expressed as mean ± standard deviation (SD). Statistical analyses were performed with GraphPad Prism software (version 9.0; GraphPad Software Inc., San Diego, CA, USA). Cell viability, apoptosis, cell-cycle distribution, ΔΨm, intracellular ROS, fluorescence image analysis, and relative gene expression data were analysed using a consistent statistical approach.

Comparisons among multiple experimental groups were carried out using one-way analysis of variance (ANOVA), followed by Tukey’s multiple-comparison post hoc test. Relative gene expression levels obtained by RT-qPCR were calculated using the 2^−ΔΔCt^ method prior to statistical evaluation.

Dose–response curves and IC_50_ values were determined by nonlinear regression analysis. Pharmacological interactions between RA and DOX were evaluated according to the Chou–Talalay median-effect principle using CompuSyn software (version 1.0; ComboSyn Inc., Paramus, NJ, USA). CI and DRI values were descriptively interpreted to characterise the nature of the pharmacological interaction and were not subjected to inferential statistical analysis.

A value of *p* < 0.05 was considered statistically significant. Statistical significance in the figures is indicated as follows: *p* < 0.05 (*), *p* < 0.01 (**), and *p* < 0.001 (***). For experiments involving NAC pretreatment, comparisons with the corresponding treatment groups are indicated as # (* *p* < 0.05), ## (** *p* < 0.01), and ### (*** *p* < 0.001).

## 3. Results

### 3.1. Concentration-Dependent Cytotoxic Effects of RA and DOX

**Figure 1:**
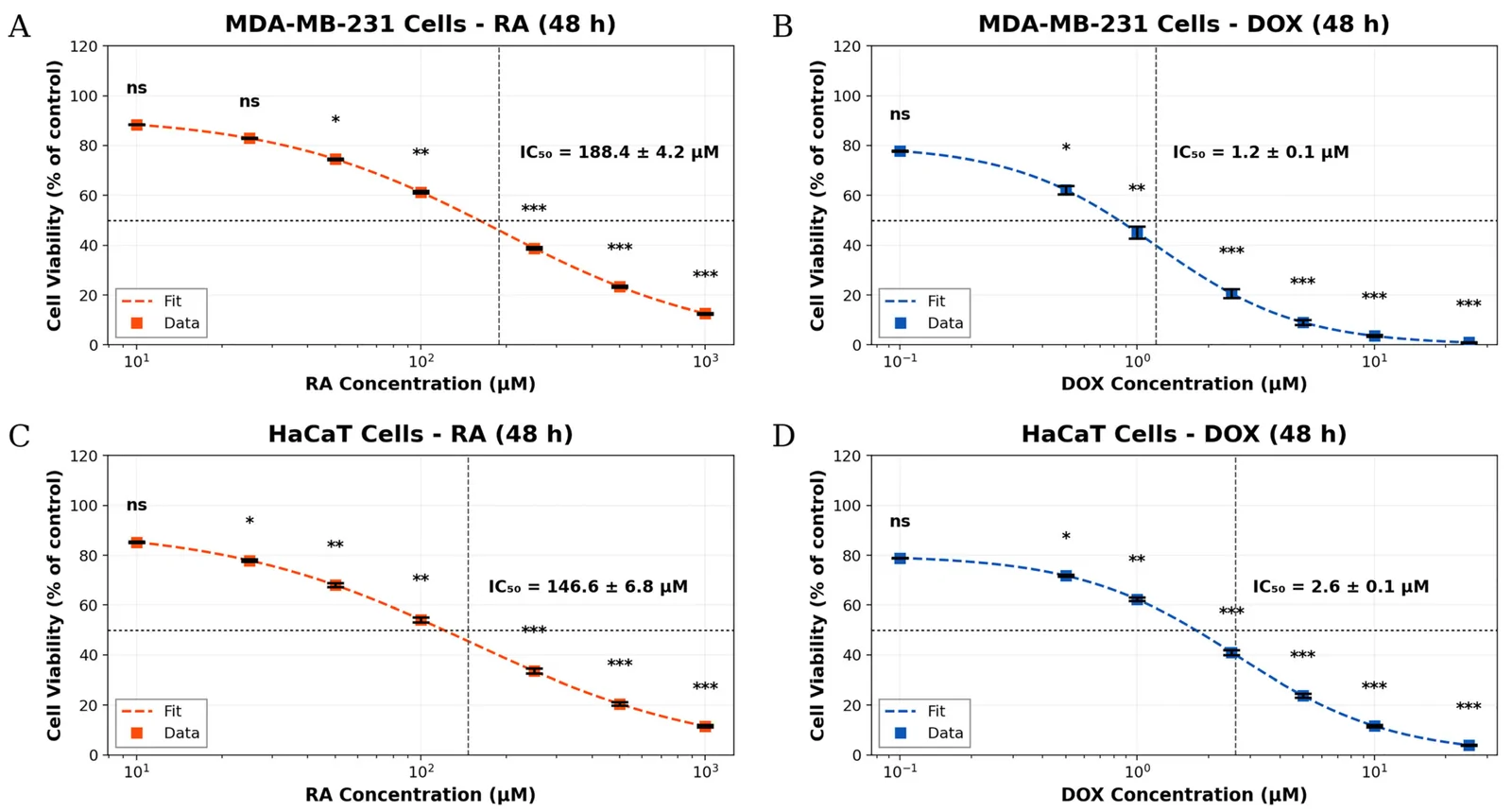
Concentration-dependent effects of RA and DOX on the viability of MDA-MB-231 and HaCaT cells after 48 h of treatment. (**A**) Dose–response curve showing the effect of RA on MDA-MB-231 cell viability and the corresponding IC_50_ value (188.4 ± 4.2 µM). (**B**) Dose–response curve illustrating the cytotoxic activity of DOX in MDA-MB-231 cells with an IC_50_ value of 1.2 ± 0.1 µM. (**C**) Dose-dependent reduction in the viability of HaCaT cells following RA treatment, with an IC_50_ value of 146.6 ± 6.8 µM. (**D**) Dose–response curve of DOX in HaCaT cells demonstrating an IC_50_ value of 2.6 ± 0.1 µM. Cell viability was determined using the MTT assay and expressed as the percentage of untreated control cells. Symbols represent experimental data, while dashed curves indicate the nonlinear regression model used for the determination of IC_50_. The horizontal dotted line denotes 50% cell viability, and the vertical dashed line indicates the calculated IC_50_ value. Data are presented as the mean ± SD of three independent biological experiments (*n* = 3). Statistical significance was determined by one-way ANOVA followed by Tukey’s multiple-comparisons post hoc test. Differences were considered significant relative to the untreated control group (* *p* < 0.05, ** *p* < 0.01, *** *p* < 0.001; ns, not significant).

The effects of RA and DOX on the viability of MDA-MB-231 and HaCaT cells were examined after 48 h of treatment using the MTT assay (Figure 1). Both compounds produced a concentration-dependent reduction in cell viability in the two cell lines, although the magnitude of the response differed according to both the compound and the cell type.

Exposure of MDA-MB-231 cells to RA progressively reduced cell viability from approximately 88% at 10 µM to approximately 13% at 1000 µM, yielding an IC_50_ value of 188.4 ± 4.2 µM (Figure 1A). DOX exhibited markedly greater cytotoxic activity, decreasing viability from approximately 78% at 0.1 µM to approximately 1% at 25 µM, with an IC_50_ value of 1.2 ± 0.1 µM (Figure 1B).

A comparable dose-dependent response was observed in HaCaT cells. RA treatment reduced cell viability from approximately 85% at 10 µM to approximately 11% at 1000 µM, corresponding to an IC_50_ value of 146.6 ± 6.8 µM (Figure 1C). Likewise, DOX decreased cell viability from approximately 79% at 0.1 µM to approximately 4% at 25 µM and produced an IC_50_ value of 2.6 ± 0.1 µM (Figure 1D).

Comparison of the calculated IC_50_ values revealed different sensitivity profiles for the two cell lines. DOX displayed a greater cytotoxic potency toward MDA-MB-231 cells than HaCaT cells, as reflected by the lower IC_50_ value (1.2 ± 0.1 versus 2.6 ± 0.1 µM). In contrast, HaCaT cells exhibited greater sensitivity to RA than MDA-MB-231 cells, with IC_50_ values of 146.6 ± 6.8 µM and 188.4 ± 4.2 µM, respectively. These concentration-response analyses established the experimental concentrations subsequently used for combination and mechanistic investigations. The corresponding replicate-level numerical data are provided in Supplementary Table S1.

### 3.2. Pharmacological Interaction Between RA and DOX

**Figure 2:**
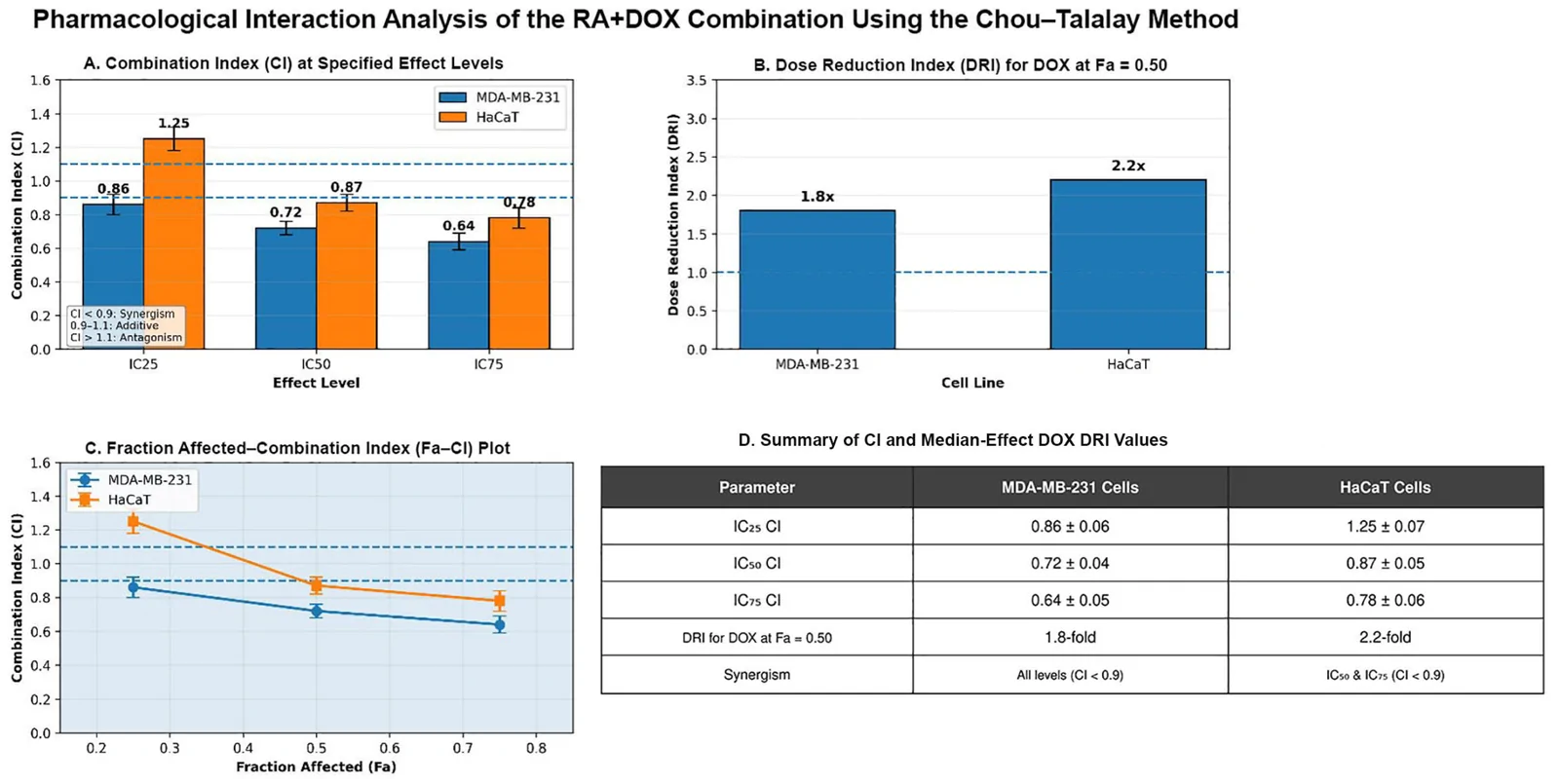
Pharmacological interaction between RA and DOX determined using the Chou–Talalay method. Drug interactions were evaluated using a fixed RA:DOX molar ratio of 157:1 based on the respective MDA-MB-231 IC_50_ values. CI values at the specified effect levels and DOX DRI values at the median-effect level were derived from the fitted fixed-ratio concentration–response model. (**A**) CI values calculated at the IC_25_, IC_50_, and IC_75_ effect levels for MDA-MB-231 and HaCaT cells. CI values < 0.90 indicate synergism, values from 0.90 to 1.10 indicate an additive interaction, and values > 1.10 indicate antagonism. (**B**) Model-derived DRI values for DOX at Fa = 0.50 in each cell line. DRI values represent descriptive comparisons of the single-agent and combination doses predicted to produce the same effect and do not indicate clinical dose reduction. (**C**) Fa–CI plot illustrating the relationship between the fraction affected and the calculated pharmacological interaction. (**D**) Summary table presenting CI values at the specified effect levels and median-effect DOX DRI values for both cell lines. CI data are presented as the mean ± SD of three independent biological experiments (*n* = 3); DOX DRI values are presented as descriptive model-derived estimates. The dashed horizontal lines indicate the CI thresholds of 0.9 and 1.1, delineating synergistic (CI < 0.9), additive (CI = 0.9–1.1), and antagonistic (CI > 1.1) interactions.

The interaction between RA and DOX was quantitatively evaluated using the Chou–Talalay median-effect method (Figure 2). Median-effect analysis generated the median-effect parameters (Dm, m, and r) for each drug using the Chou–Talalay model. In MDA-MB-231 cells, RA exhibited a Dm of 188.4 µM, m = 1.15, and r = 0.989, whereas DOX exhibited a Dm of 1.20 µM, m = 1.22, and r = 0.992. In HaCaT cells, the corresponding median-effect parameters were Dm = 285.0 µM, m = 1.08, and r = 0.985 for RA, and Dm = 2.60 µM, m = 1.16, and r = 0.988 for DOX. The HaCaT RA Dm value obtained from the linearised median-effect model (285.0 µM) differed from the IC_50_ value derived by four-parameter nonlinear regression in GraphPad Prism (146.6 µM). This difference reflects the use of distinct curve-fitting approaches: the GraphPad IC_50_ was estimated from the nonlinear concentration–response curve, whereas the CompuSyn Dm was derived from the log-transformed median-effect equation. The two estimates were therefore not treated as interchangeable, and the HaCaT Dm value was used only as a model-specific parameter within the Chou–Talalay analysis and not as an independent estimate of cytotoxic potency. In MDA-MB-231 cells, the combination produced synergistic interactions at all examined effect levels, with CI values of 0.86 ± 0.06, 0.72 ± 0.04, and 0.64 ± 0.05 at IC_25_, IC_50_, and IC_75_, respectively (Figure 2A). The progressive decline in CI values with increasing effect levels indicated a stronger pharmacological interaction as the fraction of affected cells increased.

In HaCaT cells, the interaction profile differed at low treatment efficacy. The CI value at IC_25_ was 1.25 ± 0.07, indicating antagonism, while the CI values decreased to 0.87 ± 0.05 and 0.78 ± 0.06 at IC_50_ and IC_75_, respectively, indicating synergistic interactions at moderate and high levels of effect (Figure 2A). This trend was also reflected in the Fa-CI graph, where the CI values gradually decreased with increasing Fa, particularly in MDA-MB-231 cells (Figure 2C).

At the median-effect level (Fa = 0.50), the model-derived DRI values for DOX were 1.8 in MDA-MB-231 cells and 2.2 in HaCaT cells (Figure 2B). These values were interpreted descriptively as model-based comparisons of the single-agent and combination doses predicted to produce the same effect and not as evidence of clinical dose reduction or therapeutic benefit. A summary of the CI values and median-effect DOX DRI values for both cell lines is presented in Figure 2D. The replicate-level CI data, Fa values, fixed-ratio input concentrations, and model-derived DRI values are provided in Supplementary Table S2.

### 3.3. Evaluation of Intracellular ROS-Associated Fluorescence by DCFH-DA Imaging and NAC Rescue

**Figure 3:**
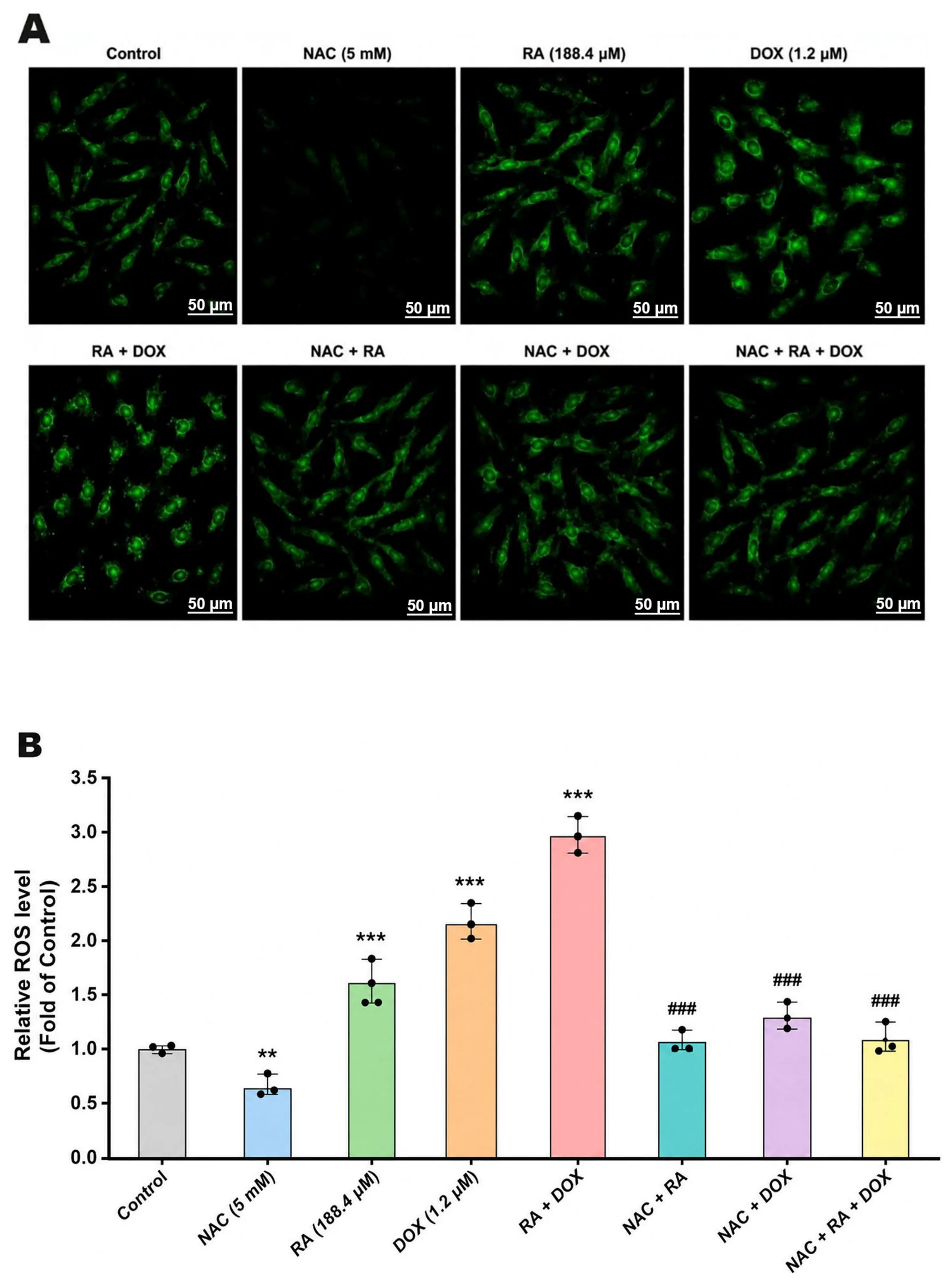
Intracellular ROS-associated fluorescence assessed using DCFH-DA in MDA-MB-231 cells after RA and DOX treatment. (**A**) Representative DCFH-DA fluorescence microscopy images of untreated control cells and cells treated with N-acetyl-L-cysteine (NAC; 5 mM), RA (188.4 µM), DOX (1.2 µM), RA + DOX, NAC + RA, NAC + DOX, and NAC + RA + DOX for 48 h. Intracellular ROS-associated fluorescence is indicated by green DCF fluorescence. The RA + DOX combination produced the highest fluorescence intensity, whereas NAC pretreatment markedly reduced intracellular ROS-associated fluorescence in all treatment groups. Scale bar = 50 µm. (**B**) Quantitative analysis of intracellular ROS-associated DCF fluorescence. Fluorescence values were normalised to the untreated control group and presented as fold change relative to the control. Data are presented as the mean ± SD of three independent biological experiments (*n* = 3). Statistical analysis was performed using one-way ANOVA followed by Tukey’s multiple-comparisons post hoc test. ** *p* < 0.01 and *** *p* < 0.001 versus the untreated control group; ### *p* < 0.001 versus the RA + DOX group.

Intracellular ROS-associated fluorescence was evaluated by DCFH-DA imaging in MDA-MB-231 cells after treatment with RA, DOX, and their combination in the presence or absence of NAC (Figure 3). Representative fluorescence images demonstrated low basal intracellular DCF fluorescence in untreated control cells, whereas NAC treatment alone further reduced the DCF fluorescence intensity. Treatment with RA or DOX alone increased intracellular ROS-associated fluorescence compared to the untreated control group. The combination of RA + DOX produced the strongest fluorescence signal, indicating the highest level of intracellular ROS-associated DCF fluorescence. In contrast, NAC pretreatment markedly attenuated intracellular fluorescence in all treatment groups, including the combination treatment, resulting in fluorescence intensities close to those observed in untreated control cells (Figure 3A).

Quantitative analysis of DCF fluorescence intensity confirmed the microscopic observations (Figure 3B). Relative intracellular DCFH-DA fluorescence levels increased to 1.6 ± 0.1 times after RA treatment and 2.1 ± 0.1 times after DOX treatment, while the combination of RA + DOX produced the highest fluorescence level (2.9 ± 0.2 times) compared to the untreated control group (** *p* < 0.01 and *** *p* < 0.001). Pretreatment with NAC significantly reduced intracellular DCFH-DA fluorescence in the RA-, DOX- and RA + DOX-treated groups to approximately basal levels, supporting effective attenuation of treatment-induced intracellular oxidative changes (Figure 3B). The replicate-level numerical data corresponding to Figure 3 are provided in Supplementary Table S3.

### 3.4. Cytometric Flow Evaluation of Apoptosis

**Figure 4:**
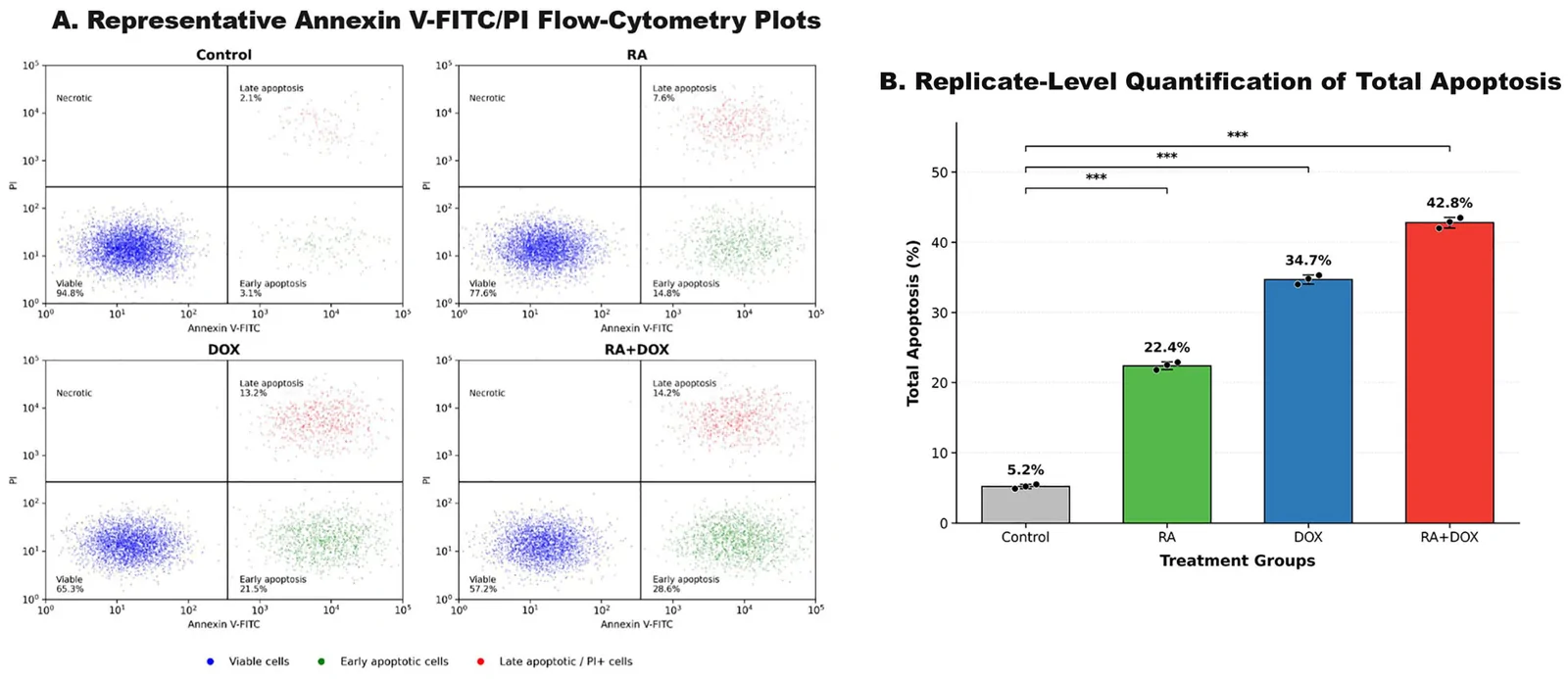
Annexin V-FITC/PI flow-cytometric analysis of apoptosis in MDA-MB-231 cells following 48 h treatment with RA (188.4 µM), DOX (1.2 µM), or their combination (188.4 µM RA + 1.2 µM DOX). (**A**) Representative Annexin V-FITC/PI dot plots. Cell populations were classified as viable (Annexin V^−^/PI^−^), early apoptotic (Annexin V^+^/PI^−^), late apoptotic/PI-positive (Annexin V^+^/PI^+^), and necrotic (Annexin V^−^/PI^+^). (**B**) Replicate-level quantification of total apoptosis, calculated as the sum of the early- and late-apoptotic populations. Total apoptosis increased from 5.2 ± 0.3% in control cells to 22.4 ± 0.6%, 34.7 ± 0.7%, and 42.8 ± 0.8% following RA, DOX, and RA + DOX treatment, respectively. Bars represent the mean ± SD of three independent biological experiments (*n* = 3), and individual points represent the values obtained in each experiment. Statistical analysis was performed using one-way ANOVA followed by Tukey’s multiple-comparisons test. Comparisons are shown relative to the untreated control (*** *p* < 0.001).

Annexin V-FITC/PI flow cytometric analysis demonstrated that treatment with RA and DOX increased apoptotic cell death in MDA-MB-231 cells, with the greatest effect observed after combination treatment (Figure 4). The proportion of viable cells progressively declined from 94.8% in the control group to 77.6%, 65.3%, and 57.2% following treatment with RA, DOX, and RA + DOX, respectively. Conversely, the percentage of early apoptotic cells (Annexin V^+^/PI^−^) increased from 3.1% in untreated cells to 14.8%, 21.5%, and 28.6% after RA, DOX, and combination treatment, respectively. Similarly, late apoptotic cells (Annexin V^+^/PI^+^) increased from 2.1% in the control group to 7.6%, 13.2%, and 14.2%, respectively. Accordingly, the total apoptotic population (early plus late apoptosis) increased from 5.2% in untreated cells to 22.4%, 34.7%, and 42.8% following RA, DOX, and RA + DOX treatment, respectively. Representative dot plots revealed a progressive redistribution of cells from the viable quadrant toward the early and late apoptotic quadrants, with the most pronounced shift observed in the combination treatment group (Figure 4). The replicate-level apoptosis data corresponding to Figure 4 are provided in Supplementary Table S4. The flow-cytometric gating strategy used for this analysis is presented in Supplementary Figure S1.

### 3.5. Cell-Cycle Distribution

**Figure 5:**
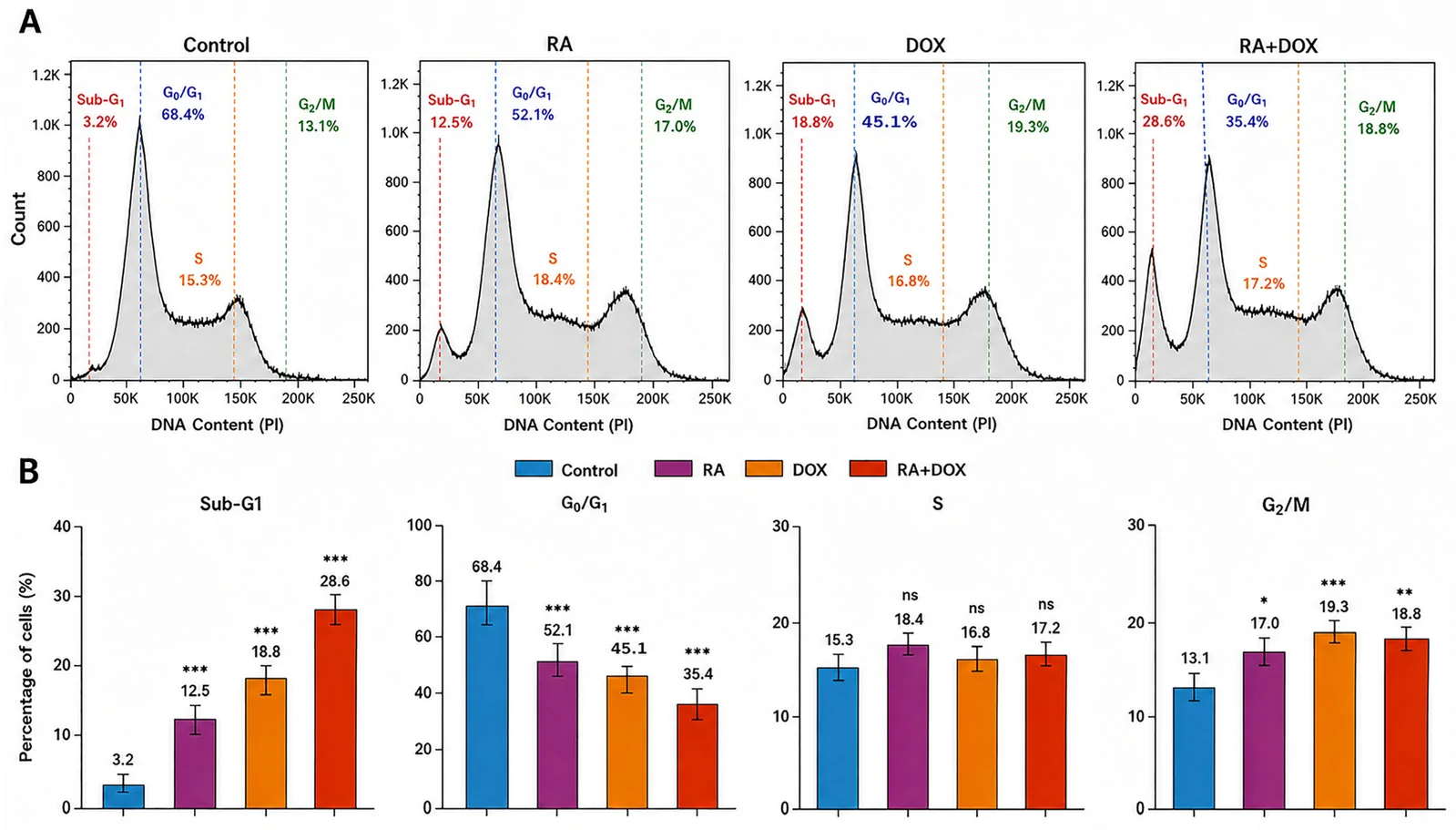
Cell-cycle distribution of MDA-MB-231 cells following 48 h treatment with RA (188.4 µM), DOX (1.2 µM), or their combination (RA + DOX; 188.4 µM RA + 1.2 µM DOX). (**A**) Representative PI flow-cytometry histograms illustrating DNA-content profiles and the distribution of cells in the Sub-G**_1_**, G**_0_**/G**_1_**, S, and G**_2_**/M phases. (**B**) Quantitative analysis of the percentage of cells in each cell-cycle phase. In the RA-treated group, the corrected Sub-G_1_, G_0_/G_1_, S, and G_2_/M distributions were 12.5%, 52.1%, 18.4%, and 17.0%, respectively, corresponding to a total of 100.0%. Data are presented as the mean ± SD of three independent biological experiments (*n* = 3). Statistical analysis was performed using one-way ANOVA followed by Tukey’s multiple-comparisons post hoc test. Statistical comparisons were made with the untreated control group (* *p* < 0.05, ** *p* < 0.01, *** *p* < 0.001; ns, not significant).

Flow cytometric cell-cycle analysis demonstrated that treatment with RA and DOX altered cell-cycle distribution in MDA-MB-231 cells, with the greatest changes observed after combination treatment (Figure 5). The proportion of cells in the Sub-G_1_ phase increased from 3.2 ± 0.4% in the control group to 12.5 ± 1.1%, 18.8 ± 1.5%, and 28.6 ± 2.0% following treatment with RA, DOX, and RA + DOX, respectively. In parallel, the G_0_/G_1_ population progressively declined from 68.4 ± 2.1% in untreated cells to 52.1 ± 1.8%, 45.1 ± 2.2%, and 35.4 ± 2.5% in the RA-, DOX-, and RA + DOX-treated groups, respectively. In contrast, the percentage of cells in the G_2_/M phase increased from 13.1 ± 0.9% in the control group to 17.0 ± 1.3%, 19.3 ± 1.1%, and 18.8 ± 1.2% following RA, DOX, and combination treatment, respectively. No statistically significant differences were observed in the S-phase population among the experimental groups (15.3 ± 1.2%, 18.4 ± 1.5%, 16.8 ± 1.4%, and 17.2 ± 1.3%, respectively). Representative DNA-content histograms showed a progressive redistribution of cells characterized by expansion of the Sub-G_1_ fraction and depletion of the G_0_/G_1_ population, with the most pronounced changes observed in the RA + DOX-treated group (Figure 5). The replicate-level cell-cycle distribution data corresponding to Figure 5 are provided in Supplementary Table S5.

### 3.6. Mitochondrial Membrane Potential (ΔΨm)

**Figure 6:**
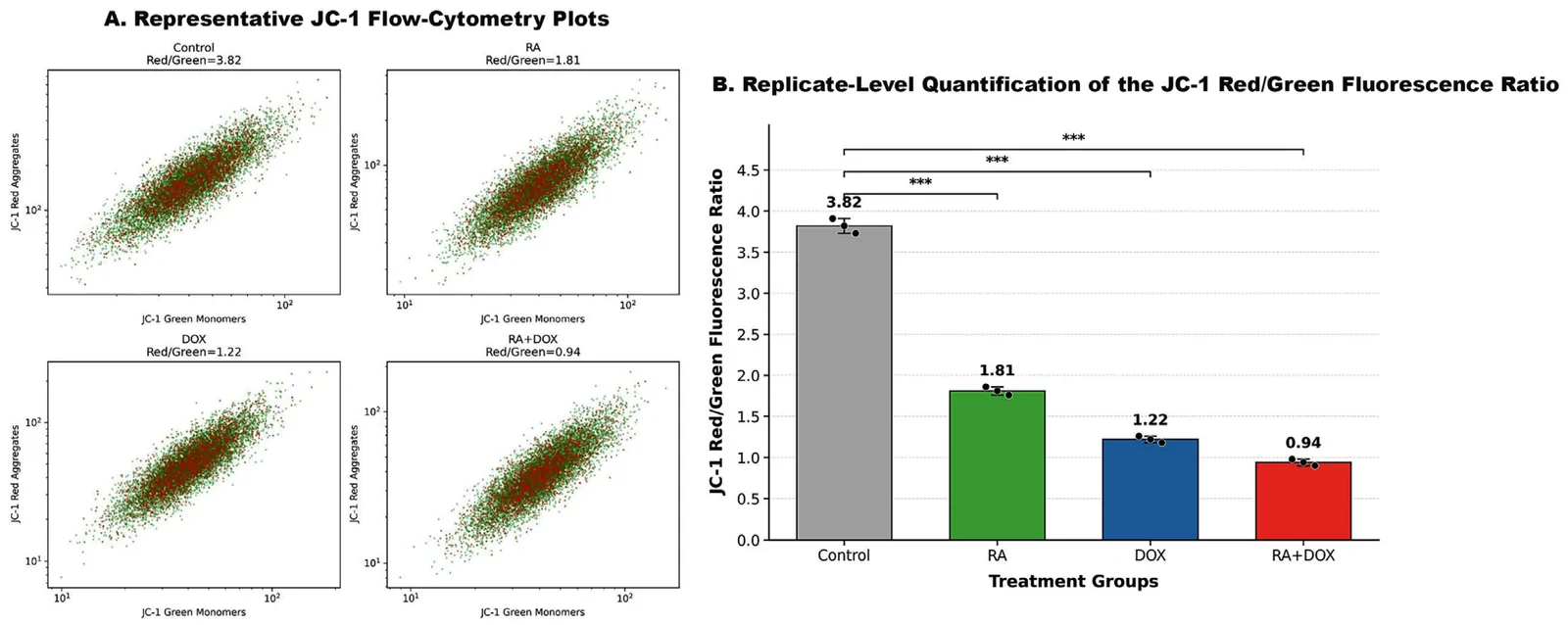
JC-1 flow-cytometric analysis of mitochondrial membrane potential (ΔΨm) in MDA-MB-231 cells following 48 h treatment with RA (188.4 µM), DOX (1.2 µM), or their combination (188.4 µM RA + 1.2 µM DOX). (**A**) Representative JC-1 flow-cytometry plots generated from 10,000 acquired events per experimental group. JC-1 red fluorescence represents mitochondrial JC-1 aggregates associated with preserved membrane potential, whereas green fluorescence represents JC-1 monomers associated with mitochondrial membrane depolarisation. (**B**) Replicate-level quantification of the JC-1 red/green fluorescence ratio. The ratio decreased from 3.82 ± 0.09 in control cells to 1.81 ± 0.05, 1.22 ± 0.04, and 0.94 ± 0.04 following RA, DOX, and RA + DOX treatment, respectively. Bars represent the mean ± SD of three independent biological experiments (*n* = 3), and individual points represent values obtained in each experiment. Statistical analysis was performed using one-way ANOVA followed by Tukey’s multiple-comparisons test. Comparisons are shown relative to the untreated control (*** *p* < 0.001).

JC-1 flow cytometric analysis demonstrated that treatment with RA and DOX reduced ΔΨm in MDA-MB-231 cells, with the greatest reduction observed after combination treatment (Figure 6). The untreated control group exhibited a high JC-1 red/green fluorescence ratio (3.82), indicating predominantly polarised mitochondria. Treatment with RA decreased the red/green fluorescence ratio to 1.81, whereas DOX treatment further reduced the ratio to 1.22. The lowest red/green fluorescence ratio was observed in the RA + DOX group (0.94), indicating the greatest loss of mitochondrial membrane potential. Representative JC-1 dot plots showed a progressive shift from red fluorescent aggregates to green fluorescent monomers after treatment, with the most pronounced shift observed in the combination group (Figure 6). The replicate-level JC-1 red/green fluorescence ratio data corresponding to Figure 6 are provided in Supplementary Table S6.

### 3.7. Cleaved Caspase-9-Associated Immunoreactivity

**Figure 7:**
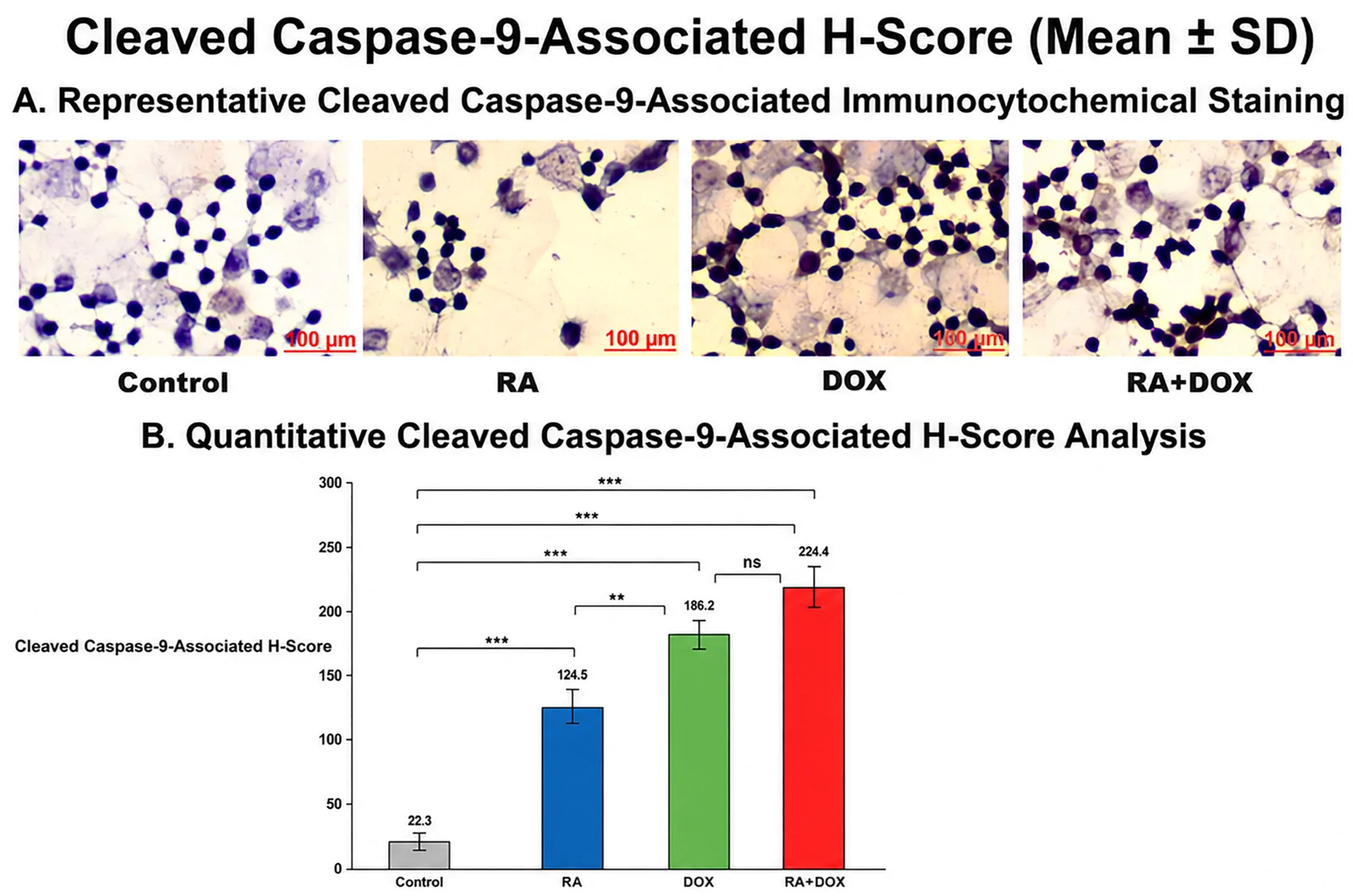
Immunocytochemical evaluation of cleaved Caspase-9-associated immunoreactivity in MDA-MB-231 cells after 48 h of treatment with RA (188.4 µM), DOX (1.2 µM), and their combination (RA + DOX; 188.4 µM RA + 1.2 µM DOX). (**A**) Representative immunocytochemical images showing cleaved Caspase-9-associated immunoreactivity in untreated control cells and treated groups. Immunoreactive cells were identified by brown cytoplasmic DAB staining, whereas the nuclei were counterstained with hematoxylin. Weak immunoreactivity was observed in the control group, while RA and DOX treatments increased cytoplasmic immunoreactivity. The strongest immunoreactivity was detected in the RA + DOX combination group. These findings were interpreted as treatment-associated differences in cleaved Caspase-9-associated immunoreactivity rather than definitive evidence of Caspase-9 activation. The images were acquired using a light microscope at 100× magnification. Scale bar = 100 µm. (**B**) Quantitative analysis of cleaved Caspase-9-associated immunoreactivity using the H-score method. H-score values increased from 22.3 ± 8.7 in the control group to 124.5 ± 12.4 after RA treatment, 186.2 ± 15.8 after DOX treatment, and 224.4 ± 22.6 after combination treatment. Data are presented as the mean ± SD of three independent biological experiments (*n* = 3). Statistical analysis was performed using one-way ANOVA followed by Tukey’s multiple-comparisons post hoc test. Statistical significance for the pairwise comparisons indicated by brackets is shown as ** *p* < 0.01, and *** *p* < 0.001; ns, not significant.

Immunocytochemical analysis demonstrated increased cleaved Caspase-9-associated immunoreactivity in MDA-MB-231 cells following RA and DOX treatment, with the strongest immunoreactivity observed in the combination group (Figure 7A,B). The untreated control group exhibited weak cytoplasmic DAB staining, corresponding to an H-score of 22.3 ± 8.7. RA treatment significantly increased cleaved Caspase-9-associated immunoreactivity, resulting in an H-score of 124.5 ± 12.4. A greater increase was observed following DOX treatment, with an H-score of 186.2 ± 15.8, whereas the highest H-score was detected in the RA + DOX group (224.4 ± 22.6; Figure 7B).

Representative immunocytochemical images demonstrated progressively stronger cytoplasmic DAB staining after RA and DOX treatment, with the most intense staining observed in the combination group (Figure 7A). In parallel, treated cells exhibited reduced cell density, cellular shrinkage, and condensed cellular morphology compared to the untreated control group. The replicate-level H-score data corresponding to Figure 7 are provided in Supplementary Table S7.

### 3.8. Nuclear Morphological Assessment by NucBlue Staining

**Figure 8:**
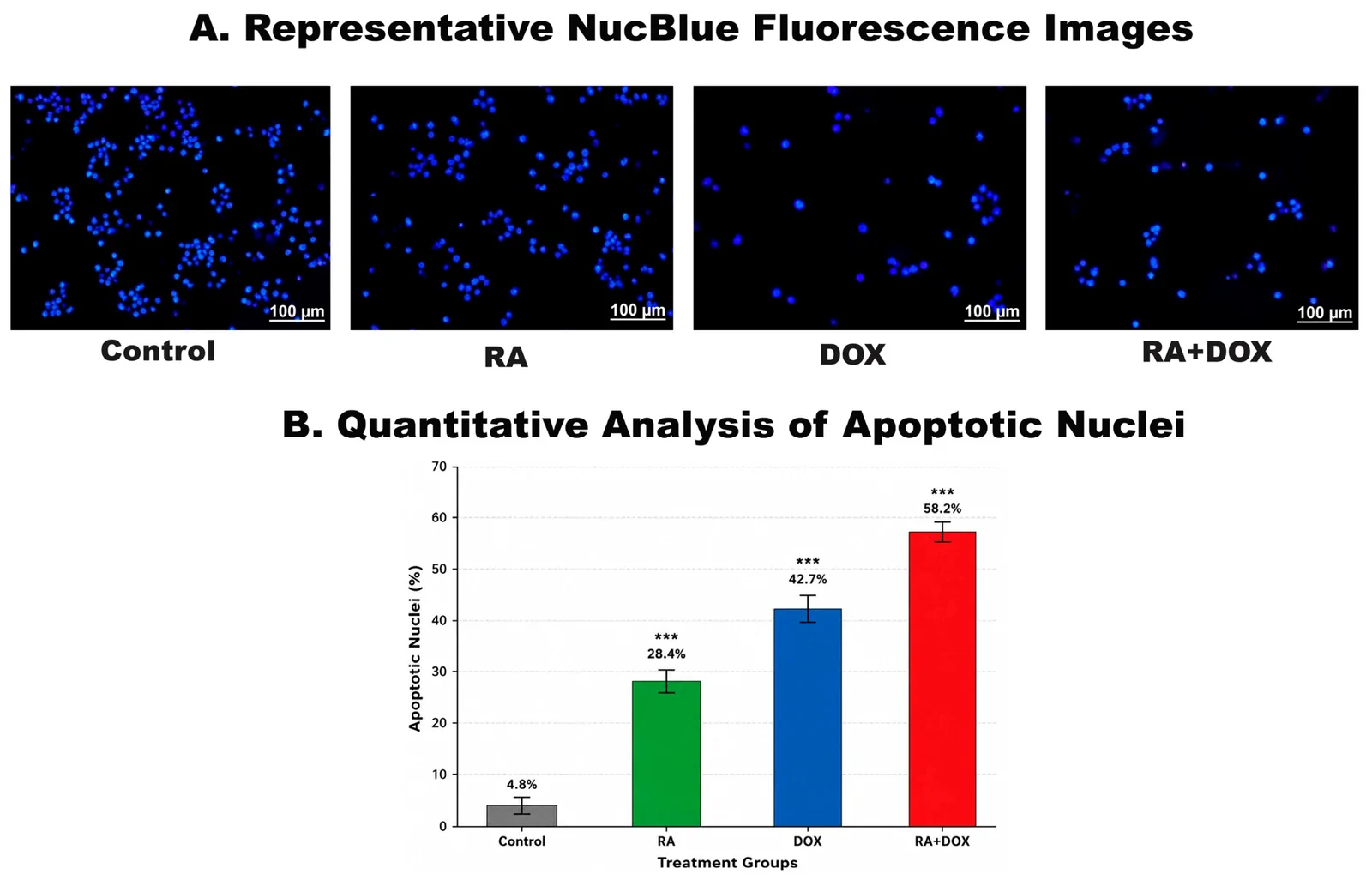
Nuclear morphological assessment of MDA-MB-231 cells following 48 h treatment with RA (188.4 µM), DOX (1.2 µM), and their combination (RA + DOX; 188.4 µM RA + 1.2 µM DOX) (**A**) Representative NucBlue fluorescence images showing nuclear morphology in untreated control cells and treated groups. Cell nuclei are visualized by blue fluorescence. Untreated control cells exhibited uniformly stained nuclei with regular morphology and high cellular density. Treatment with RA and DOX reduced cell density and increased nuclear condensation, while the combination of RA + DOX produced the most pronounced nuclear condensation and nuclear fragmentation, consistent with apoptotic cell death. The images were acquired using a fluorescence microscope at 100× magnification. Scale bar = 100 µm. (**B**) Quantitative analysis of apoptotic nuclear morphology. The percentage of nuclei exhibiting apoptotic features, including chromatin condensation and nuclear fragmentation, was determined following NucBlue staining. Data are presented as the mean ± SD of three independent biological experiments (*n* = 3). Statistical analysis was performed using one-way ANOVA followed by Tukey’s multiple-comparisons post hoc test. *** *p* < 0.001 versus the untreated control group.

NucBlue fluorescence staining was performed to evaluate treatment-induced changes in nuclear morphology in MDA-MB-231 cells (Figure 8A). Untreated control cells showed a high density of uniformly stained nuclei with regular oval morphology and a homogeneous nuclear distribution. RA treatment resulted in a moderate reduction in cell density along with the appearance of brighter and more condensed nuclei in a subset of cells. Following DOX treatment, a further decrease in cell number was observed, accompanied by increased nuclear condensation and reduced intercellular density. The most pronounced nuclear alterations were detected in the RA + DOX group, which exhibited the lowest cell density together with numerous small, intensely stained nuclei showing chromatin condensation and nuclear fragmentation. Representative fluorescence images demonstrated progressively greater nuclear morphological alterations from the RA and DOX groups to the combination treatment group (Figure 8A).

Quantitative analysis confirmed these morphological observations (Figure 8B). The percentage of cells that exhibit chromatin condensation and nuclear fragmentation increased from 4.8 ± to 1.2% in the untreated control group to 28.4 ± 3.1% following RA treatment and 42.7 ± 3.8% following DOX treatment (both *p* < 0.001 versus the control group). The highest proportion of apoptotic nuclei was observed in the RA + DOX group (58.2 ± 3.8%), representing a significant increase compared with the untreated control group (*p* < 0.001). These findings demonstrate that the combination treatment induced the most extensive apoptotic nuclear alterations, consistent with the enhanced apoptotic response observed in the Annexin V/PI, JC-1, and Caspase-9 analyses (Figure 8B). The replicate-level apoptotic nuclear morphology data corresponding to Figure 8 are provided in Supplementary Table S8.

### 3.9. qRT-PCR Analysis of Apoptosis- and Proliferation-Related Gene Expression

**Figure 9:**
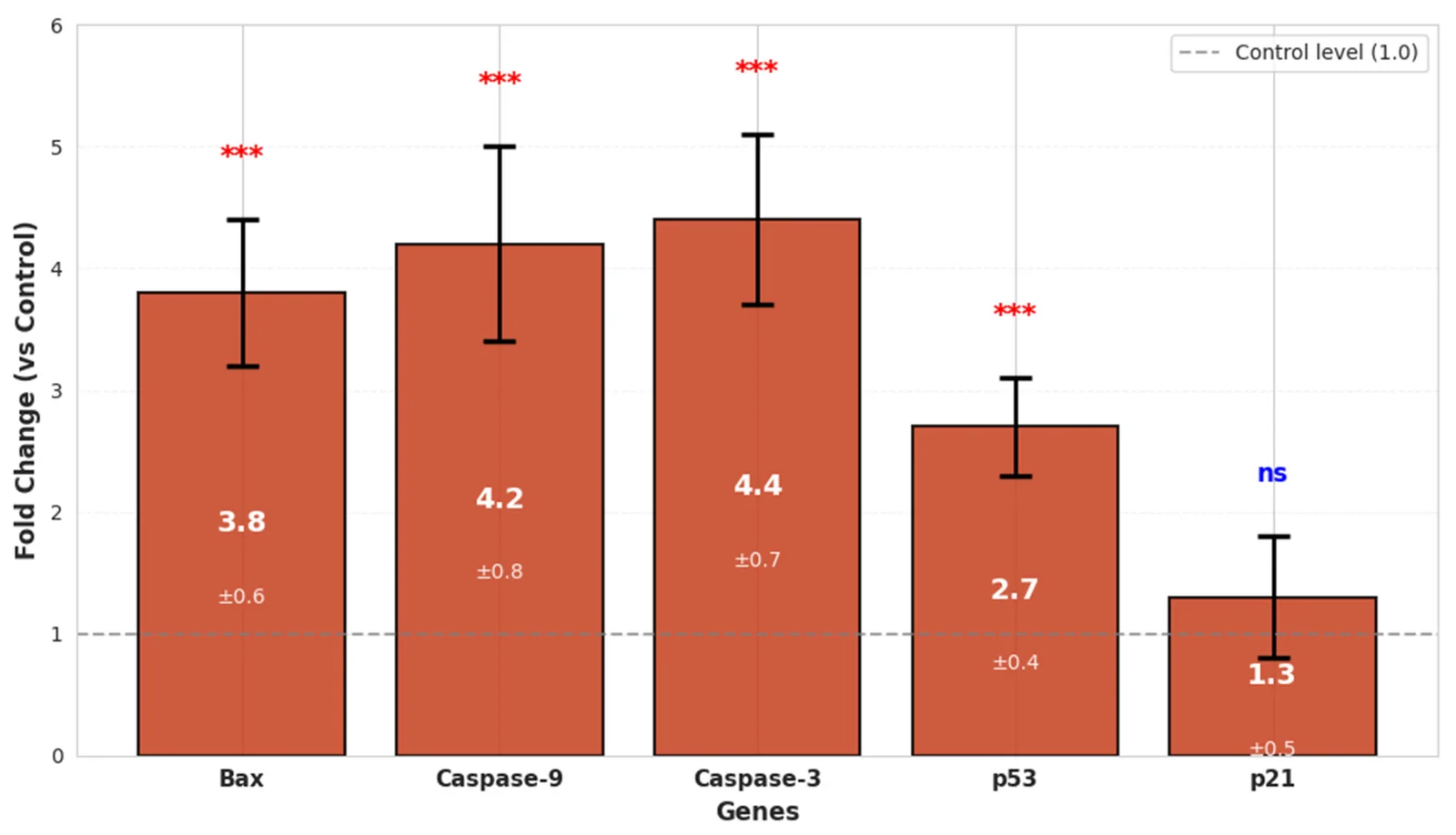
Relative expression of apoptosis-related genes in MDA-MB-231 cells after 48 h of treatment with RA (188.4 µM), DOX (1.2 µM), and their combination (RA + DOX; 188.4 µM RA + 1.2 µM DOX). Gene expression levels were normalised to the untreated control group and are presented as a fold change relative to the control. The combination of RA + DOX significantly increased the expression of *BAX*, *CASP9*, *CASP3*, and *TP53*, while the expression of *CDKN1A* (*p21*) showed a modest and non-significant increase. Data are presented as the mean ± SD of three independent biological experiments (*n* = 3). The dashed horizontal line indicates the normalised control expression level (fold change = 1.0). *** *p* < 0.001 versus the untreated control group; ns, not significant.

**Figure 10:**
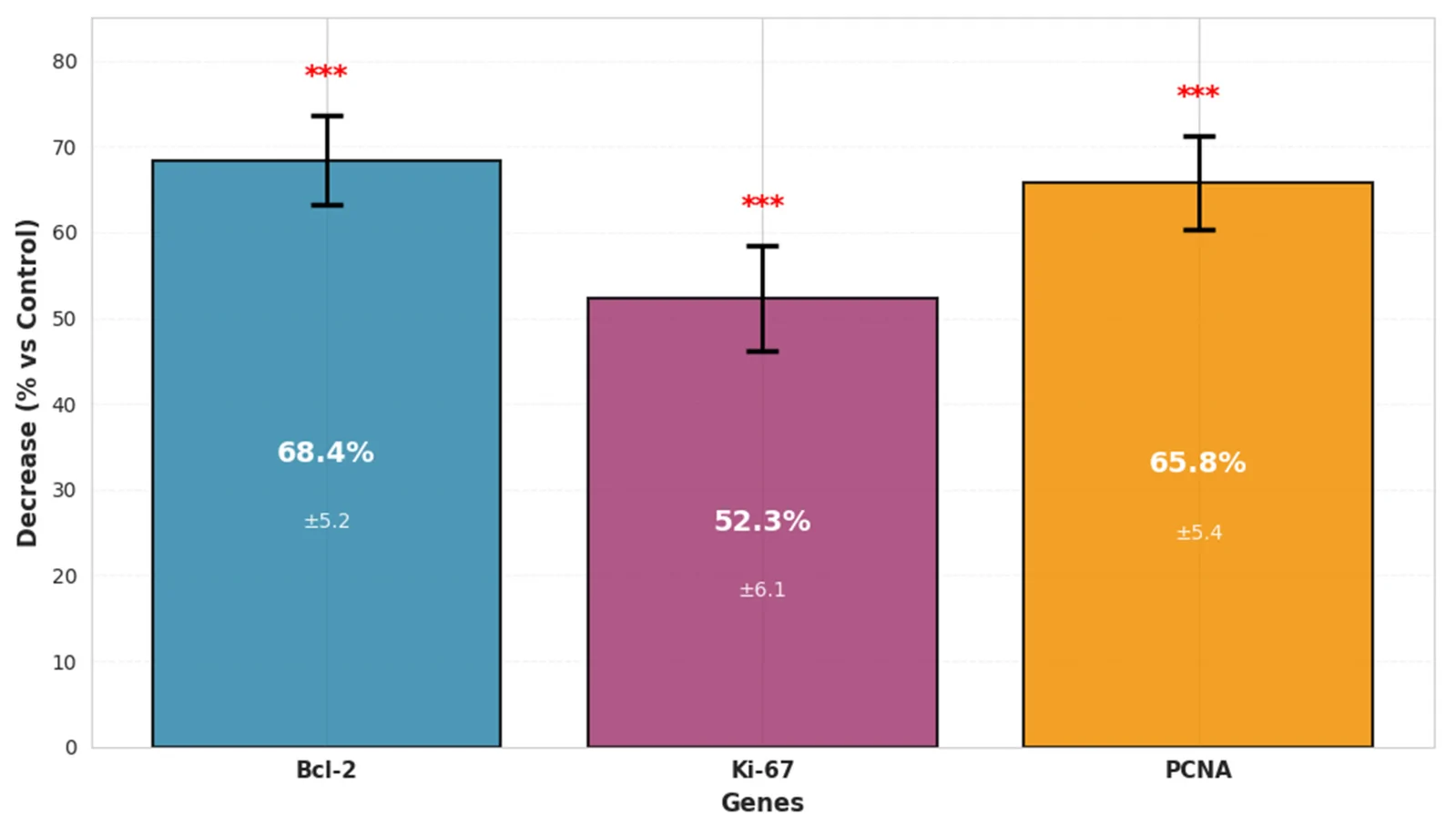
Relative expression of anti-apoptotic and proliferation-associated genes mRNA in MDA-MB-231 cells after 48 h of treatment with RA (188.4 µM), DOX (1.2 µM), and their combination (RA + DOX; 188.4 µM RA + 1.2 µM DOX). Gene expression is presented as the percentage decrease relative to the untreated control group. The combination of RA + DOX significantly reduced the expression of *BCL2*, *MKI67*, and *PCNA*. Data are presented as the mean ± SD of three independent biological experiments (*n* = 3). *** *p* < 0.001 versus the untreated control group.

RT-qPCR analysis showed that the combination RA + DOX markedly altered the expression of genes associated with apoptosis and cellular proliferation in MDA-MB-231 cells (Figure 9 and Figure 10). Relative to the untreated control group, expression of the pro-apoptotic genes *BAX*, *CASP9*, *CASP3*, and *TP53* increased by 3.8 ± 0.6-fold, 4.2 ± 0.8-fold, 4.4 ± 0.7-fold, and 2.7 ± 0.4-fold, respectively (*p* < 0.001 for all comparisons). Expression of *CDKN1A* (*p21*) increased by 1.3 ± 0.5-fold; however, this change did not reach statistical significance (Figure 9).

In contrast, the expression of the anti-apoptotic gene BCL2 decreased by 68.4 ± 5.2%, whereas expression of the proliferation-associated genes MKI67 and PCNA was reduced by 52.3 ± 6.1% and 65.8 ± 5.4%, respectively (all *p* < 0.001 versus the untreated control group) (Figure 10). The replicate-level RT-qPCR data corresponding to Figure 9 and Figure 10 are provided in Supplementary Table S9.

### 3.10. Bioinformatic Analysis of Apoptosis-Related Gene Networks

**Figure 11:**
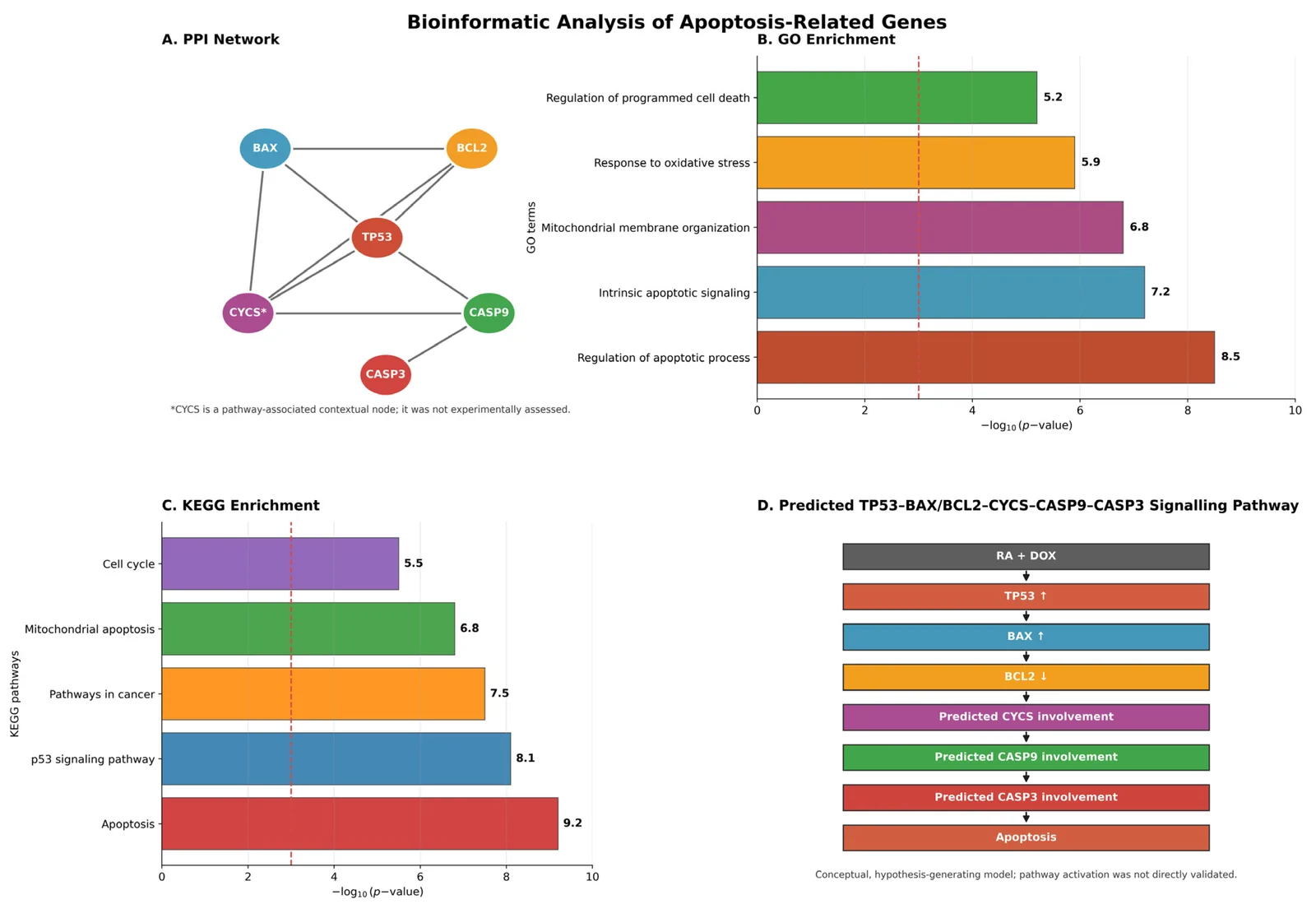
Bioinformatic analysis of apoptosis-related genes associated with RA + DOX treatment. (**A**) PPI network generated using STRING based on the experimentally assessed genes *TP53*, *BAX*, *BCL2*, *CASP9*, and *CASP3*, together with *CYCS* as a pathway-associated contextual node. *CYCS* expression and cytochrome-c release were not experimentally evaluated. (**B**) GO enrichment analysis showing the principal biological processes associated with the selected gene set. (**C**) KEGG enrichment analysis identifying the significantly enriched signalling pathways. (**D**) Conceptual representation of the predicted TP53–BAX/BCL2–CYCS–CASP9–CASP3 pathway. The bioinformatic findings are hypothesis-generating and do not constitute experimental validation of *CYCS* expression, cytochrome-c release, or pathway activation. The red dashed lines in panels B and C indicate the significance threshold [−log_10_(p) = 3]. The white arrows in panel D indicate the predicted directional relationships among pathway components.

Bioinformatic analysis was performed to provide functional context for the experimentally assessed apoptosis-related genes rather than to serve as independent mechanistic validation (Figure 11). The PPI network identified predicted interactions among *TP53*, *BAX*, *BCL2*, *CASP9*, and *CASP3*, with *TP53* occupying a central position (Figure 11A). *CYCS* was included only as a pathway-associated contextual node and was not evaluated by RT-qPCR or any cytochrome-c release assay.

GO enrichment analysis demonstrated significant enrichment of biological processes associated with regulation of programmed cell death, intrinsic apoptotic signaling, mitochondrial membrane organization, and cellular responses to oxidative stress (Figure 11B). KEGG pathway enrichment analysis further identified apoptosis, the p53 signaling pathway, pathways in cancer, mitochondrial apoptosis, and cell cycle regulation as the most significantly enriched pathways (Figure 11C). These analyses should be interpreted as a complementary functional overview of the selected apoptosis-related genes rather than as an unbiased genome-wide enrichment analysis. Integration of these analyses generated a predicted conceptual model connecting the experimentally assessed genes with the contextual *CYCS* node within the TP53–BAX/BCL2–CYCS–CASP9–CASP3 pathway (Figure 11D).

## 4. Discussion

The present study shows that combined RA and DOX exposure produces increased cytotoxic activity in MDA-MB-231 cells, accompanied by pharmacological synergism, increased intracellular oxidative activity, mitochondrial membrane depolarisation, apoptosis, and alterations in proliferation-associated signalling. Unlike previous investigations that focused on isolated biological endpoints, the present study integrates complementary experimental approaches to characterise multiple cellular responses associated with the RA + DOX combination [23,24]. These findings are consistent with the broader concept that naturally derived polyphenols may modify cellular responses to conventional chemotherapy through effects on multiple pathways involved in cancer progression, treatment resistance, and apoptotic cell death [2,3,4,25,26,27,28].

One of the main findings of the present study was that the increased cytotoxic response to combined RA and DOX exposure was accompanied by changes associated with intracellular oxidative stress, mitochondrial dysfunction, and apoptosis. The combination produced the greatest increase in intracellular DCFH-DA fluorescence, which was attenuated by NAC pretreatment. NAC also partially restored cell viability, supporting a contributory role of intracellular oxidative stress in treatment-associated cytotoxicity. However, because oxidative activity was evaluated only at the 48 h endpoint and NAC rescue was not assessed for apoptosis or mitochondrial membrane depolarisation, these findings do not establish oxidative stress as an upstream cause of mitochondrial dysfunction or apoptosis. Increased DCFH-DA fluorescence coincided with loss of mitochondrial membrane potential, enhanced Caspase-9 immunoreactivity, an increased proportion of Annexin V-positive cells, and chromatin condensation and nuclear fragmentation detected by NucBlue staining. These cellular findings were accompanied by increased mRNA expression of *BAX*, *CASP9*, *CASP3*, and *TP53*, together with decreased *BCL2* mRNA expression and a reduced *BCL2/BAX* mRNA expression ratio. Bioinformatic analyses further identified predicted interactions among apoptosis- and oxidative stress-associated genes. Collectively, these findings are consistent with the involvement of intracellular oxidative stress, mitochondrial dysfunction, and apoptotic processes in the response to combined RA and DOX exposure; however, they do not demonstrate a direct causal sequence or establish activation of the intrinsic mitochondrial apoptotic pathway [21,22,29,30].

The increased apoptotic response observed after combined RA and DOX exposure coincided with increased intracellular DCFH-DA fluorescence. Attenuation of the DCFH-DA signal and partial restoration of cell viability following NAC pretreatment suggest that intracellular oxidative stress contributed to treatment-associated cytotoxicity. However, DCFH-DA detects general intracellular oxidative activity and is not specific for mitochondrial ROS; therefore, the present findings do not identify the source of the oxidative signal or establish its temporal relationship with mitochondrial depolarisation and apoptosis. Although RA is widely recognised for its antioxidant properties under physiological conditions, previous evidence indicates that it may exhibit context-dependent pro-oxidant activity in cancer cells [31,32]. DOX is also known to induce intracellular oxidative stress and oxidative DNA damage [33,34]. Accordingly, the increased oxidative activity observed after combined treatment provides a plausible biological context for the accompanying mitochondrial and apoptotic alterations, but a direct causal relationship cannot be inferred from the present experiments. Zhou et al. demonstrated that MAEL promotes the progression of breast cancer by inducing chaperone-mediated autophagic degradation of citrate synthase and fumarate hydratase, facilitating a metabolic shift from oxidative phosphorylation to aerobic glycolysis. Given the close relationship between mitochondrial metabolism, intracellular ROS homeostasis, and apoptotic susceptibility, these findings provide an additional biological context supporting therapeutic strategies that target mitochondrial function and oxidative stress to improve the efficacy of conventional chemotherapy [35]. Similar ROS-associated chemosensitizing effects have been reported for several naturally occurring phenolic compounds [15,16,36,37]. However, whether such effects can improve tumour-specific activity without increasing toxicity in non-malignant cells requires compound- and model-specific experimental validation.

Recent studies have increasingly explored the use of naturally occurring polyphenols as chemosensitizing agents to enhance the therapeutic efficacy of DOX while overcoming drug resistance and reducing treatment-associated toxicity. Polyphenolic compounds including curcumin, resveratrol, quercetin, epigallocatechin gallate (EGCG), hesperidin, and RA have been reported to enhance DOX-induced cytotoxicity through multiple mechanisms, including modulation of oxidative stress, disruption of mitochondrial function, activation of apoptotic signalling, and inhibition of survival-associated pathways [2,15,16,26,27,28,38]. Recent reviews published in 2024 further identified RA as one of the most promising naturally derived chemosensitizers due to its ability to simultaneously regulate oxidative stress, inflammation, apoptosis, and multidrug resistance-related signalling networks [15,16]. The present study extends these observations by integrating complementary experimental approaches that characterise the pharmacological interaction and associated oxidative, mitochondrial, apoptotic, and molecular responses to combined RA and DOX exposure in MDA-MB-231 cells.

Recent advances in DOX-based therapy increasingly emphasise the development of combination strategies and advanced drug-delivery approaches to improve therapeutic efficacy while minimising systemic toxicity. Despite its well-established antitumor activity, the clinical application of DOX remains limited by cumulative dose-dependent cardiotoxicity, which has been associated with mitochondrial injury, oxidative stress, vascular damage, and activation of stress-responsive signalling pathways [39]. Consequently, considerable effort has been directed toward developing therapeutic strategies capable of improving antitumor efficacy while reducing the effective dose and systemic toxicity of DOX. Recent comprehensive reviews have highlighted that current research is focused on optimising DOX treatment through combination regimens, targeted therapies, and nanoformulations designed to enhance intratumoral drug accumulation, overcome drug resistance, and reduce adverse effects while improving the therapeutic index. Furthermore, nanoparticle-based delivery systems, including liposomes, polymeric micelles, and other nanocarriers, have emerged as promising platforms to increase tumour-selective drug delivery and reduce off-target toxicity [40,41]. In addition, rational molecular engineering of DOX prodrugs has enabled the development of carrier-free nanoassemblies that achieve enhanced tumour accumulation, prolonged circulation, controlled drug release, and superior antitumor efficacy, highlighting the importance of optimising both drug delivery and pharmacological performance to maximise the therapeutic benefit of DOX [42]. In parallel, ROS-responsive galactosylated nanoparticles have been shown to enable controlled DOX release within the oxidative tumour microenvironment, thus enhancing apoptosis, inducing cell-cycle arrest, promoting intratumoral drug accumulation, and suppressing tumour growth in TNBC models [43]. Similarly, multifunctional nanocarriers that integrate targeted drug delivery with radionuclide labelling have demonstrated efficient cellular internalisation, prolonged tumour retention, and enhanced therapeutic efficacy against TNBC, further illustrating the potential of nanotechnology-assisted strategies to improve DOX-based treatment [44]. Although the present study did not investigate nanoparticle-based delivery systems, future studies may examine whether alternative delivery approaches modify the cellular activity and toxicity profile of the RA + DOX combination [40,41,43].

In addition to its pro-apoptotic activity, RA has been proposed to enhance the efficacy of conventional chemotherapeutic agents by modulating mechanisms associated with multidrug resistance. Previous studies have suggested that RA may regulate ATP-binding cassette (ABC) transporters, particularly ABCB1/P-glycoprotein, thus increasing intracellular retention of anticancer drugs and improving therapeutic responsiveness [45,46]. Although this mechanism could partially contribute to the synergistic interaction observed in the present study, neither the expression of the ABC transporter nor the intracellular accumulation of DOX was experimentally evaluated. Consequently, the potential participation of multidrug resistance modulation should be regarded as a plausible, yet untested mechanism. Future studies incorporating transporter expression analyses, intracellular drug accumulation assays, and functional inhibition experiments will be necessary to clarify whether regulation of ABC transporters contributes to the chemosensitizing effect of RA.

Because MDA-MB-231 cells harbor a mutant *TP53* genotype, the increased *TP53* mRNA expression observed following RA + DOX treatment should not be interpreted as restoration of canonical wild-type p53 tumor suppressor function. Rather, elevated expression of *TP53* is more likely to reflect cellular stress responses triggered by oxidative damage, mitochondrial dysfunction, and DNA damage, all of which were supported by increased intracellular ROS levels, mitochondrial membrane depolarisation, and enhanced apoptotic activity observed in the present study. Similarly, the modest increase in *CDKN1A* (*p21*) expression may represent the activation of stress-responsive cell-cycle regulatory mechanisms that operate through both the p53-dependent and the p53-independent pathways. Bioinformatic analysis also identified the p53 signalling pathway as one of the significantly enriched pathways; however, pathway enrichment alone does not establish functional activation. Therefore, although the present findings are consistent with the activation of DNA damage-associated signalling networks, additional protein-based investigations, including the evaluation of mutant p53 activity, p21 protein expression, and downstream signalling events, will be required to clarify the biological significance of the regulation of *TP53* and *CDKN1A* in this TNBC model. Comparison of MDA-MB-231 and HaCaT cells revealed cell model-dependent differences in treatment response but did not establish tumour selectivity. Although the RA + DOX combination produced a lower magnitude of cytotoxicity in HaCaT keratinocytes than in MDA-MB-231 cells under the examined conditions, HaCaT cells were more sensitive to RA alone, as indicated by the respective IC_50_ values, and the RA + DOX combination also showed synergistic interactions in HaCaT cells at moderate and high effect levels. Differences in redox regulation and mitochondrial homeostasis between malignant and non-malignant cells may influence their responses to anticancer treatments [12,15,16]; however, ROS production, mitochondrial membrane potential, and apoptosis were not comparatively evaluated in HaCaT cells in the present study. Therefore, the observed cell line-dependent responses cannot be attributed to differences in redox homeostasis and should not be interpreted as evidence of preferential tumour-cell sensitisation, tumour-selective activity, or an improved therapeutic index. Furthermore, because HaCaT keratinocytes are not derived from mammary epithelium, validation in non-malignant mammary epithelial cells, additional TNBC models, and appropriate in vivo systems is required before the selectivity, safety, or translational relevance of the RA + DOX combination can be determined.

Bioinformatic analyses provided hypothesis-generating information regarding potential functional relationships among apoptosis-associated genes and predicted pathways related to the combined treatment. Protein–protein interaction analysis positioned *TP53* as a central node within a predicted network comprising *BAX*, *BCL2*, *CYCS*, *CASP9*, and *CASP3*, whereas GO and KEGG enrichment analyses identified apoptosis, the p53 signalling pathway, oxidative stress-related processes, and cancer-associated pathways among the enriched functional categories [47]. *CYCS* was included only as a predicted network-associated gene and was not experimentally evaluated by RT-qPCR in the present study. Although some predicted associations were consistent with the experimentally observed transcriptional changes, mitochondrial membrane depolarisation, increased intracellular oxidative activity, and enhanced Caspase-9 immunoreactivity, the bioinformatic analyses do not demonstrate pathway activation or functional involvement of the predicted proteins. These computational findings should therefore be regarded as hypothesis-generating and require confirmation using targeted protein-based and functional experiments.

The present study further expands on our previous investigations exploring the chemosensitizing potential of RA in combination with conventional chemotherapeutic agents in different cancer models. In earlier studies, RA improved the antitumor efficacy of gemcitabine, cisplatin, and DOX in ovarian carcinoma and retinoblastoma cells through the promotion of apoptosis and the modulation of survival-associated signalling pathways [3,4,12,17,19]. The current findings extend these observations to the highly aggressive triple-negative breast cancer model while providing a substantially broader mechanistic characterisation of the underlying biological responses. By integrating pharmacological interaction analysis, intracellular oxidative activity assessment with NAC modulation, mitochondrial membrane potential analysis, Annexin V/PI apoptosis, NucBlue-based nuclear morphology, Caspase-9 immunocytochemistry, apoptosis- and proliferation-related gene-expression profiling, and bioinformatic pathway analysis, the present study provides a multidimensional characterisation of the cellular responses associated with combined RA and DOX exposure [48,49]. The concordance among these experimental endpoints indicates that increased intracellular oxidative activity, mitochondrial membrane depolarisation, and apoptosis occurred concurrently; however, it does not establish oxidative stress-dependent activation of mitochondrial apoptosis or a common causal mechanism across different tumour types.

Several limitations of the present study should be considered when interpreting the findings. First, all experiments were conducted under in vitro conditions using only one triple-negative breast cancer cell line (MDA-MB-231). Although MDA-MB-231 cells represent a well-established model of aggressive TNBC, the use of a single TNBC cell line limits the generalizability of the present findings. Therefore, validation in additional TNBC models, as well as hormone receptor-positive and HER2-positive breast cancer cell lines, would improve the generalizability of the present findings. Second, while apoptosis- and proliferation-related gene expression was comprehensively characterized by RT-qPCR, the corresponding changes at the protein level were not confirmed using Western blotting or other quantitative protein-based approaches. Likewise, assessment of post-translational modifications and activation states of key signaling molecules would provide a more comprehensive understanding of the molecular mechanisms underlying the observed biological responses. Third, although the DCFH-DA assay together with NAC modulation experiments supported a contributory role of intracellular oxidative stress in treatment-associated cytotoxicity, DCFH-DA detects general intracellular oxidative activity and does not specifically measure mitochondrial ROS. Intracellular oxidative activity was assessed only at the 48 h endpoint, and NAC rescue was evaluated for cell viability but not for apoptosis or mitochondrial membrane depolarisation. Therefore, the present experiments do not establish the temporal or causal relationships among oxidative stress, mitochondrial dysfunction, and apoptosis or determine whether the observed mitochondrial alterations were associated with mitochondria-derived ROS. Future studies should incorporate early time-course measurements, mitochondria-specific ROS probes such as MitoSOX Red, and NAC rescue analyses of apoptosis and mitochondrial membrane potential, together with assessments of antioxidant enzyme activity and oxidative DNA damage. In addition, protein-level assessment of the BAX/BCL2 ratio, cleaved caspase-3 and Caspase-9, PARP cleavage, and mitochondrial cytochrome c release will be required to determine whether the intrinsic mitochondrial apoptotic pathway is functionally activated. Fourth, the predicted signaling pathways identified by bioinformatic analyses were not functionally validated using targeted pathway inhibition or gene-silencing approaches. Finally, the pharmacokinetic profile, biodistribution, systemic toxicity, and therapeutic efficacy of the RA + DOX combination remain to be established in appropriate orthotopic or xenograft breast cancer models before consideration of clinical translation. An additional limitation concerns the evaluation of treatment selectivity. Although HaCaT keratinocytes were included as a non-malignant reference cell line, they do not represent normal mammary epithelial cells. Furthermore, HaCaT cells exhibited greater sensitivity to RA than MDA-MB-231 cells, and synergistic interactions were also observed following the RA + DOX combination. Therefore, the present findings do not establish tumour-selective chemosensitization or an improved therapeutic index. Future studies should validate these findings using non-malignant mammary epithelial cell models, such as MCF10A, together with additional TNBC cell lines, to more comprehensively evaluate treatment selectivity and translational potential.

## 5. Conclusions

The present study shows that combined RA and DOX exposure produces synergistic cytotoxic activity in MDA-MB-231 cells, accompanied by increased intracellular oxidative activity, mitochondrial membrane depolarisation, apoptosis, cell-cycle perturbation, and changes in proliferation-associated gene expression. NAC pretreatment attenuated the treatment-associated increase in DCFH-DA fluorescence and partially restored cell viability, supporting a contributory role of intracellular oxidative stress in cytotoxicity. Increased intracellular oxidative activity coincided with mitochondrial membrane depolarisation, enhanced apoptotic cell death, increased Caspase-9 immunoreactivity, nuclear condensation and fragmentation, changes in apoptosis- and proliferation-related mRNA expression, and predicted enrichment of apoptosis-associated pathways in bioinformatic analyses. However, these findings do not establish mitochondrial ROS generation, a direct causal relationship between oxidative stress and mitochondrial dysfunction, or functional activation of the intrinsic mitochondrial apoptotic pathway.

Collectively, the pharmacological, cellular, transcriptional, immunocytochemical, and computational findings provide an integrated description of the responses associated with combined RA and DOX exposure rather than definitive evidence of a specific causal mechanism. The HaCaT comparison provided limited cytotoxicity data in a non-malignant, non-mammary cell model but was not sufficient to evaluate tumour-selective chemosensitization, treatment safety, or improvement of the therapeutic index. Accordingly, the present findings should be regarded as an exploratory in vitro basis for further investigation of the RA + DOX combination. Validation using additional TNBC cell lines, non-malignant mammary epithelial models, other breast cancer subtypes, and appropriate in vivo systems is required to determine the reproducibility, selectivity, safety, and potential translational relevance of the observed responses.

## Figures and Tables

**Table 1:** Primer sequences used for RT-qPCR analysis.

Gene	Forward Primer Sequence (5′–3′)	Reverse Primer Sequence (5′–3′)
*BAX*	CCCGAGAGGTCTTTTTCCGAG	CCAGCCCATGATGGTTCTGAT
*BCL2*	GGTGGGGTCATGTGTGTGG	CGGTTCAGGTACTCAGTCATCC
*CASP3*	CATGGAAGCGAATCAATGGACT	CTGTACCAGACCGAGATGTCA
*CASP9*	CTCAGACCAGAGATTCGCAAAC	GCATTTCCCCTCAAACTCTCAA
*TP53*	CAGCACATGACGGAGGTTGT	TCATCCAAATACTCCACACGC
*CDKN1A*	TGTCCGTCAGAACCCATGC	AAAGTCGAAGTTCCATCGCTC
*MKI67*	ACGCCTGGTTACTATCAAAAGG	CAGACCCATTTACTTGTGTTGGA
*PCNA*	CCTGCTGGGATATTAGCTCCA	CAGCGGTAGGTGTCGAAGC
*GAPDH*	GGAGCGAGATCCCTCCAAAAT	GGCTGTTGTCATACTTCTCATGG
*ACTB*	CATGTACGTTGCTATCCAGGC	CTCCTTAATGTCACGCACGAT

## Data Availability

The numerical source data underlying the quantitative analyses are provided in the Supplementary Materials. Raw flow-cytometry (.fcs) files are available from the corresponding author upon reasonable request.

## Supplementary Materials

The following supporting information can be downloaded at: https://www.mdpi.com/article/10.3390/biomedicines14091977/s1, Figure S1: Flow-cytometric gating strategy and representative Annexin V-FITC/PI apoptosis analysis in MDA-MB-231 cells; Table S1: Numerical data corresponding to Figure 1; Table S2: Raw numerical data corresponding to Figure 2; Table S3: Numerical data corresponding to Figure 3; Table S4: Numerical data corresponding to Figure 4; Table S5: Numerical data corresponding to Figure 5; Table S6: Numerical data corresponding to Figure 6; Table S7: Numerical data corresponding to Figure 7; Table S8: Numerical data corresponding to Figure 8; Table S9: Numerical data corresponding to Figure 9 and Figure 10.

## Abbreviations

The following abbreviations are used in this manuscript:

ABC	ATP-Binding Cassette
ANOVA	Analysis of Variance
CI	Combination Index
DAB	3,3′-Diaminobenzidine
DCFH-DA	2′,7′-Dichlorodihydrofluorescein Diacetate
DMSO	Dimethyl Sulfoxide
DOX	Doxorubicin
DRI	Dose Reduction Index
EDTA	Ethylenediaminetetraacetic Acid
FITC	Fluorescein Isothiocyanate
FSC	Forward Scatter
GO	Gene Ontology
IC_50_	Half-Maximal Inhibitory Concentration
JC-1	5,5′,6,6′-Tetrachloro-1,1′,3,3′-Tetraethylbenzimidazolylcarbocyanine Iodide
KEGG	Kyoto Encyclopedia of Genes and Genomes
MFI	Mean Fluorescence Intensity
MTT	3-(4,5-Dimethylthiazol-2-yl)-2,5-Diphenyltetrazolium Bromide
NAC	N-Acetyl-L-Cysteine
PBS	Phosphate-Buffered Saline
PI	Propidium Iodide
PPI	Protein–Protein Interaction
RA	Rosmarinic Acid
ROS	Reactive Oxygen Species
RT-qPCR	Reverse Transcription Quantitative Polymerase Chain Reaction
SD	Standard Deviation
SSC	Side Scatter
STRING	Search Tool for the Retrieval of Interacting Genes/Proteins
TNBC	Triple-Negative Breast Cancer
ΔΨm	Mitochondrial Membrane Potential
