# Supplementary material for: Rosmarinic Acid Enhances Doxorubicin Activity in MDA-MB-231 Cells: Associations with Intracellular Oxidative Stress, Mitochondrial Dysfunction, and Apoptosis
Source: Biomedicines. 2026 Sep 2;14(9):1977. doi: 10.3390/biomedicines14091977 (PMC13604353; doi:10.3390/biomedicines14091977)
Supplement: Supplementary file 1 [file biomedicines-14-01977-s001.zip › Tables S1-S9.pdf]

Table S1. Numerical data corresponding to Figure 1.

Figure 1 panel	Cell line	Treatment	Concentration (μM)	Replicate 1 (%)	Replicate 2 (%)	Replicate 3 (%)	Mean ± SD (%)
**A**	MDA-MB-231	RA	10	88.32	88.40	88.48	**88.40 ± 0.08**
**A**	MDA-MB-231	RA	25	82.70	82.91	83.10	**82.90 ± 0.20**
**A**	MDA-MB-231	RA	50	74.21	74.56	74.90	**74.56 ± 0.35**
**A**	MDA-MB-231	RA	100	60.82	61.33	61.82	**61.32 ± 0.50**
**A**	MDA-MB-231	RA	250	38.28	38.83	39.38	**38.83 ± 0.55**
**A**	MDA-MB-231	RA	500	22.94	23.37	23.80	**23.37 ± 0.43**
**A**	MDA-MB-231	RA	1000	12.33	12.60	12.87	**12.60 ± 0.27**
**B**	MDA-MB-231	DOX	0.1	77.51	77.79	78.02	**77.78 ± 0.26**
**B**	MDA-MB-231	DOX	0.5	60.52	62.30	63.84	**62.22 ± 1.66**
**B**	MDA-MB-231	DOX	1.0	42.72	45.20	47.45	**45.12 ± 2.36**
**B**	MDA-MB-231	DOX	2.5	18.76	20.62	22.43	**20.61 ± 1.84**
**B**	MDA-MB-231	DOX	5.0	8.12	9.08	10.05	**9.08 ± 0.97**
**B**	MDA-MB-231	DOX	10	3.20	3.60	4.02	**3.61 ± 0.41**
**B**	MDA-MB-231	DOX	25	0.88	1.00	1.12	**1.00 ± 0.12**
**C**	HaCaT	RA	10	85.03	85.29	85.53	**85.28 ± 0.25**
**C**	HaCaT	RA	25	77.36	77.90	78.41	**77.89 ± 0.52**
**C**	HaCaT	RA	50	67.18	68.02	68.79	**68.00 ± 0.80**
**C**	HaCaT	RA	100	53.12	54.17	55.17	**54.16 ± 1.03**
**C**	HaCaT	RA	250	32.54	33.55	34.52	**33.54 ± 0.99**
**C**	HaCaT	RA	500	19.73	20.48	21.21	**20.47 ± 0.74**
**C**	HaCaT	RA	1000	11.02	11.49	11.96	**11.49 ± 0.47**

Figure 1 panel	Cell line	Treatment	Concentration (µM)	Replicate 1 (%)	Replicate 2 (%)	Replicate 3 (%)	Mean ± SD (%)
**D**	HaCaT	DOX	0.1	78.84	78.90	78.95	**78.90 ± 0.05**
**D**	HaCaT	DOX	0.5	71.41	71.80	72.15	**71.79 ± 0.37**
**D**	HaCaT	DOX	1.0	61.57	62.29	62.97	**62.28 ± 0.70**
**D**	HaCaT	DOX	2.5	40.00	41.03	42.03	**41.02 ± 1.01**
**D**	HaCaT	DOX	5.0	22.92	23.78	24.61	**23.77 ± 0.85**
**D**	HaCaT	DOX	10	11.12	11.62	12.12	**11.62 ± 0.50**
**D**	HaCaT	DOX	25	3.70	3.89	4.07	**3.89 ± 0.19**

Table S1. Numerical data corresponding to Figure 1. Cell viability values for MDA-MB-231 and HaCaT cells following 48 h exposure to the indicated concentrations of rosmarinic acid (RA) or doxorubicin (DOX). Replicates 1–3 represent three independent biological experiments (*n* = 3). Cell viability was normalized to the untreated control and expressed as a percentage of control viability. Data are summarized as mean ± SD.

Table S2. Raw numerical data corresponding to Figure 2.

Figure 2 panel	Cell line	Parameter / Effect level	RA (µM)	DOX (µM)	Fa	CI Replicate 1	CI Replicate 2	CI Replicate 3	CI Mean ± SD	DOX DRI
**A, C**	MDA-MB-231	IC_{25}	—	—	0.25	0.80	0.86	0.92	**0.86 ± 0.06**	—
**A, C**	MDA-MB-231	IC_{50}	—	—	0.50	0.68	0.72	0.76	**0.72 ± 0.04**	—
**A, C**	MDA-MB-231	IC_{75}	—	—	0.75	0.59	0.64	0.69	**0.64 ± 0.05**	—
**A, C**	HaCaT	IC_{25}	—	—	0.25	1.18	1.25	1.32	**1.25 ± 0.07**	—
**A, C**	HaCaT	IC_{50}	—	—	0.50	0.82	0.87	0.92	**0.87 ± 0.05**	—
**A, C**	HaCaT	IC_{75}	—	—	0.75	0.72	0.78	0.84	**0.78 ± 0.06**	—
**B**	MDA-MB-231	DRI at median effect	—	—	0.50	—	—	—	—	**1.8**
**B**	HaCaT	DRI at median effect	—	—	0.50	—	—	—	—	**2.2**
**Analysis input**	Both cell lines	Fixed-ratio dose 1	47.1	0.30	—	—	—	—	—	—
**Analysis input**	Both cell lines	Fixed-ratio dose 2	94.2	0.60	—	—	—	—	—	—
**Analysis input**	Both cell lines	Fixed-ratio dose 3	188.4	1.20	—	—	—	—	—	—
**Analysis input**	Both cell lines	Fixed-ratio dose 4	376.8	2.40	—	—	—	—	—	—
**Analysis input**	Both cell lines	Fixed-ratio dose 5	753.6	4.80	—	—	—	—	—	—

Table S2. Raw numerical data corresponding to the pharmacological interaction analysis presented in Figure 2. Individual CI values from three independent biological experiments (Replicates 1–3) are provided together with the corresponding mean ± SD values for MDA-MB-231 and HaCaT cells. Fa values of 0.25, 0.50, and 0.75 correspond to the IC_{25}, IC_{50}, and IC_{75} effect levels, respectively. The RA+DOX combination was evaluated using a fixed RA:DOX molar ratio of 157:1. DOX DRI values at Fa = 0.50 were 1.8 for MDA-MB-231 cells and 2.2 for HaCaT cells. Panels A and C are represented by the individual CI and Fa data, Panel B by the DOX DRI values, and Panel D summarizes the numerical results presented in Panels A–C and therefore is not duplicated in this table.

Table S3. Numerical data corresponding to Figure 3.

Figure 3 panel	Treatment group	Replicate 1	Replicate 2	Replicate 3	Mean ± SD (fold of control)
**B**	Control	0.95	1.00	1.05	**1.00 ± 0.05**
**B**	NAC (5 mM)	0.50	0.60	0.70	**0.60 ± 0.10**
**B**	RA (188.4 μM)	1.48	1.58	1.72	**1.59 ± 0.12**
**B**	DOX (1.2 μM)	2.00	2.10	2.20	**2.10 ± 0.10**
**B**	RA + DOX	2.80	2.95	3.10	**2.95 ± 0.15**
**B**	NAC + RA	1.00	1.08	1.18	**1.09 ± 0.09**
**B**	NAC + DOX	1.18	1.26	1.36	**1.27 ± 0.09**
**B**	NAC + RA + DOX	0.98	1.08	1.17	**1.08 ± 0.10**

Table S3. Numerical data corresponding to Figure 3. Intracellular ROS-associated DCF fluorescence values in MDA-MB-231 cells following 48 h treatment with NAC (5 mM), rosmarinic acid (RA; 188.4 μM), doxorubicin (DOX; 1.2 μM), RA + DOX, NAC + RA, NAC + DOX, or NAC + RA + DOX. Replicates 1–3 represent three independent biological experiments (*n* = 3). Fluorescence values were normalized to the untreated control and expressed as fold change relative to the control. Data are summarized as mean ± SD. Panel A contains the representative DCFH-DA fluorescence images, whereas the numerical data presented here correspond to the quantitative analysis in Panel B.

Table S4. Numerical data corresponding to Figure 4

Figure 4 panel	Treatment group	Parameter	Replicate 1 (%)	Replicate 2 (%)	Replicate 3 (%)	Mean ± SD (%)
**A**	Control	Viable cells	95.1	94.8	94.5	**94.8 ± 0.3**
**A**	Control	Early apoptosis	2.9	3.1	3.3	**3.1 ± 0.2**
**A**	Control	Late apoptosis	2.0	2.1	2.2	**2.1 ± 0.1**
**B**	Control	Total apoptosis	4.9	5.2	5.5	**5.2 ± 0.3**
**A**	RA (188.4 μM)	Viable cells	78.2	77.6	77.0	**77.6 ± 0.6**
**A**	RA (188.4 μM)	Early apoptosis	14.4	14.8	15.2	**14.8 ± 0.4**
**A**	RA (188.4 μM)	Late apoptosis	7.4	7.6	7.8	**7.6 ± 0.2**
**B**	RA (188.4 μM)	Total apoptosis	21.8	22.4	23.0	**22.4 ± 0.6**
**A**	DOX (1.2 μM)	Viable cells	66.0	65.3	64.6	**65.3 ± 0.7**
**A**	DOX (1.2 μM)	Early apoptosis	21.1	21.5	21.9	**21.5 ± 0.4**
**A**	DOX (1.2 μM)	Late apoptosis	12.9	13.2	13.5	**13.2 ± 0.3**
**B**	DOX (1.2 μM)	Total apoptosis	34.0	34.7	35.4	**34.7 ± 0.7**
**A**	RA + DOX	Viable cells	58.0	57.2	56.4	**57.2 ± 0.8**
**A**	RA + DOX	Early apoptosis	28.1	28.6	29.1	**28.6 ± 0.5**
**A**	RA + DOX	Late apoptosis	13.9	14.2	14.5	**14.2 ± 0.3**
**B**	RA + DOX	Total apoptosis	42.0	42.8	43.6	**42.8 ± 0.8**

Table S4. Numerical data corresponding to Figure 4. Annexin V-FITC/PI flow-cytometric data for MDA-MB-231 cells following 48 h treatment with rosmarinic acid (RA; 188.4 μM), doxorubicin (DOX; 1.2 μM), or their combination. Replicates 1–3 represent three independent biological experiments ($n = 3$). Viable, early apoptotic, and late apoptotic cell populations are expressed as percentages of the analyzed cells. Total apoptosis was calculated as the sum of the early- and late-apoptotic populations for each independent experiment. Data are summarized as mean ± SD. Panel A presents the representative Annexin V-FITC/PI flow-cytometry plots, whereas Panel B presents replicate-level quantification of total apoptosis.

Table S5. Numerical data corresponding to Figure 5.

Figure 5 panel	Treatment group	Cell-cycle phase	Replicate 1 (%)	Replicate 2 (%)	Replicate 3 (%)	Mean ± SD (%)
**B**	Control	Sub-G_1	3.6	2.8	3.2	**3.2 ± 0.4**
**B**	Control	G_0/G_1	69.4	69.8	66.0	**68.4 ± 2.1**
**B**	Control	S	14.5	14.7	16.7	**15.3 ± 1.2**
**B**	Control	G_2/M	12.5	12.7	14.1	**13.1 ± 0.9**
**B**	RA (188.4 μM)	Sub-G_1	13.6	11.4	12.5	**12.5 ± 1.1**
**B**	RA (188.4 μM)	G_0/G_1	54.0	52.0	50.3	**52.1 ± 1.8**
**B**	RA (188.4 μM)	S	16.7	19.6	18.8	**18.4 ± 1.5**
**B**	RA (188.4 μM)	G_2/M	15.7	17.0	18.4	**17.0 ± 1.3**
**B**	DOX (1.2 μM)	Sub-G_1	20.3	17.3	18.8	**18.8 ± 1.5**
**B**	DOX (1.2 μM)	G_0/G_1	46.0	46.7	42.6	**45.1 ± 2.2**
**B**	DOX (1.2 μM)	S	15.3	17.0	18.1	**16.8 ± 1.4**
**B**	DOX (1.2 μM)	G_2/M	18.4	19.0	20.5	**19.3 ± 1.1**
**B**	RA + DOX	Sub-G_1	30.6	26.6	28.6	**28.6 ± 2.0**
**B**	RA + DOX	G_0/G_1	35.7	37.7	32.8	**35.4 ± 2.5**
**B**	RA + DOX	S	15.9	17.2	18.5	**17.2 ± 1.3**
**B**	RA + DOX	G_2/M	17.8	18.5	20.1	**18.8 ± 1.2**

Table S5. Numerical data corresponding to Figure 5. Cell-cycle distribution values for MDA-MB-231 cells following 48 h treatment with rosmarinic acid (RA; 188.4 μM), doxorubicin (DOX; 1.2 μM), or their combination. Replicates 1–3 represent three independent biological experiments ($n = 3$). The percentages of cells in the Sub-G_1, G_0/G_1, S, and G_2/M phases are reported for each experiment, with the four cell-cycle fractions summing to 100% within each replicate. Data are summarized as mean ± SD. Panel A presents representative DNA-content histograms, whereas the numerical data presented here correspond to the quantitative cell-cycle analysis in Panel B.

Table S6. Numerical data corresponding to Figure 6.

Figure 6 panel	Treatment group	Replicate 1	Replicate 2	Replicate 3	Mean ± SD (JC-1 red/green ratio)
**B**	Control	3.73	3.82	3.91	**3.82 ± 0.09**
**B**	RA (188.4 μM)	1.76	1.81	1.86	**1.81 ± 0.05**
**B**	DOX (1.2 μM)	1.18	1.22	1.26	**1.22 ± 0.04**
**B**	RA + DOX	0.90	0.94	0.98	**0.94 ± 0.04**

Table S6. Numerical data corresponding to Figure 6. JC-1 red/green fluorescence ratios for MDA-MB-231 cells following 48 h treatment with rosmarinic acid (RA; 188.4 μM), doxorubicin (DOX; 1.2 μM), or their combination. Replicates 1–3 represent three independent biological experiments (n = 3). The JC-1 red/green fluorescence ratio was used as an indicator of mitochondrial membrane potential (ΔΨm). Data are summarized as mean ± SD. Panel A presents representative JC-1 flow-cytometry plots, whereas the numerical data presented here correspond to the replicate-level quantitative analysis in Panel B.

Table S7. Numerical data corresponding to Figure 7.

Figure 7 panel	Treatment group	Replicate 1	Replicate 2	Replicate 3	Mean ± SD (H-score)
**B**	Control	13.6	22.3	31.0	**22.3 ± 8.7**
**B**	RA (188.4 μM)	112.1	124.5	136.9	**124.5 ± 12.4**
**B**	DOX (1.2 μM)	170.4	186.2	202.0	**186.2 ± 15.8**
**B**	RA + DOX	201.8	224.4	247.0	**224.4 ± 22.6**

Table S7. Numerical data corresponding to Figure 7. Cleaved caspase-9-associated H-score values for MDA-MB-231 cells following 48 h treatment with rosmarinic acid (RA; 188.4 μM), doxorubicin (DOX; 1.2 μM), or their combination. Replicates 1–3 represent three independent biological experiments (*n* = 3). Cleaved caspase-9-associated immunoreactivity was quantified using the H-score method. Data are summarized as mean ± SD. Panel A presents representative immunocytochemical staining images, whereas the numerical data presented here correspond to the quantitative H-score analysis in Panel B.

Table S8. Numerical data corresponding to Figure 8.

**Figure 8 panel**	**Treatment group**	**Replicate 1 (%)**	**Replicate 2 (%)**	**Replicate 3 (%)**	**Mean ± SD (%)**
**B**	Control	3.6	4.8	6.0	**4.8 ± 1.2**
**B**	RA (188.4 μM)	25.3	28.4	31.5	**28.4 ± 3.1**
**B**	DOX (1.2 μM)	38.9	42.7	46.5	**42.7 ± 3.8**
**B**	RA + DOX	54.4	58.2	62.0	**58.2 ± 3.8**

Table S8. Numerical data corresponding to Figure 8. Apoptotic nuclear morphology values for MDA-MB-231 cells following 48 h treatment with rosmarinic acid (RA; 188.4 μM), doxorubicin (DOX; 1.2 μM), or their combination. Replicates 1–3 represent three independent biological experiments (n = 3). The percentage of nuclei exhibiting apoptotic morphological features, including chromatin condensation and nuclear fragmentation, was determined following NucBlue staining. Data are summarized as mean ± SD. Panel A presents representative NucBlue fluorescence images, whereas the numerical data presented here correspond to the quantitative analysis of apoptotic nuclei in Panel B.

Table S9. Numerical data corresponding to Figures 9 and 10.

Figure	Gene	Expression parameter	Replicate 1	Replicate 2	Replicate 3	Mean ± SD
**9**	*BAX*	Fold change vs. control	3.2	3.8	4.4	**3.8 ± 0.6**
**9**	*CASP9*	Fold change vs. control	3.4	4.2	5.0	**4.2 ± 0.8**
**9**	*CASP3*	Fold change vs. control	3.7	4.4	5.1	**4.4 ± 0.7**
**9**	*TP53*	Fold change vs. control	2.3	2.7	3.1	**2.7 ± 0.4**
**9**	*CDKN1A (p21)*	Fold change vs. control	0.8	1.3	1.8	**1.3 ± 0.5**
**10**	*BCL2*	Decrease vs. control (%)	63.2	68.4	73.6	**68.4 ± 5.2**
**10**	*MKI67*	Decrease vs. control (%)	46.2	52.3	58.4	**52.3 ± 6.1**
**10**	*PCNA*	Decrease vs. control (%)	60.4	65.8	71.2	**65.8 ± 5.4**

Table S9. Numerical data corresponding to Figures 9 and 10. RT-qPCR data for MDA-MB-231 cells following 48 h treatment with the combination of rosmarinic acid (RA; 188.4 μM) and doxorubicin (DOX; 1.2 μM). Replicates 1–3 represent three independent biological experiments (n = 3). Expression levels of *BAX*, *CASP9*, *CASP3*, *TP53*, and *CDKN1A (p21)* are expressed as fold change relative to the untreated control, whereas changes in *BCL2*, *MKI67*, and *PCNA* are expressed as percentage decrease relative to the untreated control. Data are summarized as mean ± SD.
